# Interleukin-38 interacts with destrin/actin-depolymerizing factor in human keratinocytes

**DOI:** 10.1371/journal.pone.0225782

**Published:** 2019-11-26

**Authors:** Dominique Talabot-Ayer, Loïc Mermoud, Julia Borowczyk, Justyna Drukala, Michal Wolnicki, Ali Modarressi, Wolf-Henning Boehncke, Nicolo Brembilla, Gaby Palmer

**Affiliations:** 1 Department of Pathology-Immunology, University of Geneva School of Medicine, Geneva, Switzerland; 2 Division of Rheumatology, Department of Internal Medicine Specialties, University Hospitals, Geneva, Switzerland; 3 Division of Dermatology and Venereology, University Hospitals, Geneva, Switzerland; 4 Cell Bank, Department of Cell Biology, Faculty of Biochemistry, Biophysics and Biotechnology, Jagiellonian University, Cracow, Poland; 5 Department of Pediatric Urology, Jagiellonian University Medical College, Cracow, Poland; 6 Department of Plastic, Reconstructive and Aesthetic Surgery, University Hospitals of Geneva, University of Geneva School of Medicine, Geneva, Switzerland; INSERM, FRANCE

## Abstract

Interleukin (IL)-38 is a member of the IL-1 family of cytokines, which was proposed to exert anti-inflammatory effects. IL-38 is constitutively expressed in the skin, where keratinocytes are the main producing cells. Little information is currently available concerning IL-38 biology. Here, we investigated the subcellular localization and interaction partners of the IL-38 protein in human keratinocytes. IL-38 expression was reduced in primary keratinocytes grown in monolayer (2D) cultures. We thus used IL-38 overexpressing immortalized normal human keratinocytes (NHK/38) to study this cytokine in cell monolayers. In parallel, differentiation of primary human keratinocytes in an *in vitro* reconstructed human epidermis (RHE) 3D model allowed us to restore endogenous IL-38 expression. In NHK/38 cells and in RHE, IL-38 was mainly cell-associated, rather than released into culture supernatants. Intracellular IL-38 was preferentially, although not exclusively, cytoplasmic. Similarly, in normal human skin sections, IL-38 was predominantly cytoplasmic in the epidermis and essentially excluded from keratinocyte nuclei. A yeast two-hybrid screen identified destrin/actin-depolymerizing factor (DSTN) as a potential IL-38-interacting molecule. Co-immunoprecipitation and proximity ligation assay confirmed this interaction. We further observed partial co-localization of IL-38 and DSTN in NHK/38 cells. Endogenous IL-38 and DSTN were also co-expressed in all epidermal layers in RHE and in normal human skin. Finally, IL-38 partially co-localized with F-actin in NHK/38 cells, in particular along the cortical actin network and in filopodia. In conclusion, IL-38 is found predominantly in the cytoplasm of human keratinocytes, where it interacts with DSTN. The functional relevance of this interaction remains to be investigated.

## Introduction

The interleukin (IL)-1 family of cytokines includes seven pro-inflammatory agonists (IL-1α IL-1β, IL-18, IL-33, IL-36α, IL-36β and IL-36γ) and four members with antagonist or anti-inflammatory activity (IL-1Ra, IL-36Ra, IL-37 and IL-38) [1]. The agonistic cytokines play essential roles in host defense, but they also cause immunopathology and their activity is controlled at many levels, including regulation of their production, maturation and release, as well as inhibition of their biological effects by decoy receptors and antagonists, which limit inflammatory responses [2, 3].

IL-38 (IL-1F10, IL-1HY2) was initially proposed to act as an IL-1 family receptor antagonist based on its sequence similarity with IL-1Ra and IL-36Ra [4]. Subsequently, anti-inflammatory effects of IL-38 were observed in cultured cells [5–9] and overexpression of IL-38 or administration of the recombinant protein attenuated disease symptoms in several mouse models of inflammation [7, 9–13]. In humans, elevated IL-38 expression or serum levels have been reported in a number of inflammatory and autoimmune diseases, with the notable exception of inflammatory skin disorders, in which IL-38 expression appears to be rather reduced [6, 9, 14–21]. In any case, a potential role of IL-38 in these conditions remains to be established. Likewise, *IL1F10* gene polymorphisms have been associated with inflammatory diseases or disease markers [22–29], but it is currently unclear whether these associations represent causal relationships.

IL-38 is constitutively expressed in the skin, where keratinocytes are the main producing cells [30]. Little information is currently available concerning subcellular localization, post-translational maturation, release or secretion of the IL-38 protein, or even potential intracellular functions of this cytokine, as suggested for other IL-1 family members [31]. In this study, we examined the localization of the IL-38 protein in human keratinocytes, which was predominantly cytoplasmic in monolayer and 3D cultures, as well as in normal human epidermis. We further identified destrin/actin-depolymerizing factor (DSTN) as an IL-38 interacting protein and observed partial co-localization of IL-38 with DSTN, as well as with F-actin, in particular along the cell cortex and in filopodia.

## Materials and methods

### Cloning of human IL-38 expression vectors

The human full-length IL-38 coding sequence (GenBank accession n° NM_173161.3, bp 76–534) was amplified by RT-PCR on total RNA extracted from cultured human primary keratinocytes using the following primers: forward 5’-AAGCTTGACACCACTGATTGCAGGAATG-3’; reverse 5’-CTCGAGGTTTCCTGTCTCCCTACCAGCTCTG-3’. The cDNA was then cloned into the pcDNA3.1(+) vector (Thermo Fisher Scientific AG) to obtain plasmid pcDNA3.1/hIL-38 for IL-38 overexpression under the control of the CMV promoter in human embryonic kidney (HEK) 293T cells or into pcDNA4/TO (Thermo Fisher Scientific AG) to obtain the plasmid pcDNA4/TO/hIL-38 for inducible IL-38 expression in an immortalized normal human keratinocyte (NHK) cell line [32]. For both constructs, the sequence of the entire coding region and of the vector-insert junctions was verified before use.

### Human skin samples

Skin biopsies were taken from healthy adults undergoing surgery at the Department of Plastic and Reconstructive Surgery of the Geneva University Hospitals in Switzerland, or children undergoing surgery at the Polish-American Children's Hospital in Cracow, Poland. This study was conducted according to the Declaration of Helsinki, and approved by the local ethics committees of the University Hospitals of Geneva (protocol 06–063), or of the Jagiellonian University in Cracow according to Polish law (No. 1072.6120.9.2017), as appropriate. Written informed consent was obtained for each individual. For histology, skin biopsies were fixed in 4% buffered formaldehyde and embedded in paraffin. For RNA extraction, the skin was rinsed in PBS containing 100 U/ml penicillin and 100 μg/ml streptomycin (P/S) and incubated in Dispase (5U/ml, STEMCELL Technologies, Vancouver, Canada) overnight at 4°C to detach the epidermis from the dermis. Both layers were then collected and frozen separately. For the isolation of primary human keratinocytes, the skin was rinsed in PBS, P/S and incubated in Dispase overnight at 4°C. The epidermis was then detached from the dermis and treated 3 times for 1 minute with 0.05% Trypsin, 0.02% EDTA. Isolated cells were cultured in Dulbecco’s modified Eagle’s medium (DMEM; Sigma-Aldrich Chemie GmbH) supplemented with 10% fetal bovine serum (Sigma-Aldrich Chemie GmbH) and P/S. After 24 h, the medium was changed to fully supplemented serum-free Keratinocyte Growth Medium 2 (KBM-2; Promocell, Heidelberg, Germany).

### Cell culture and transfection

NHK cells were cultured in keratinocyte-serum free medium (K-SFM, Thermo Fisher Scientific AG), supplemented with bovine pituitary extract and human recombinant epidermal growth factor (Thermo Fisher Scientific AG), penicillin and streptomycin, at 37°C in a humidified atmosphere containing 5% CO_2_. For doxycycline (Dox)-inducible IL-38 overexpression, we used the T-REx system (Thermo Fisher Scientific AG). NHK cells were first transfected with the pcDNA6/TR vector, which encodes the tetracycline repressor (TR) protein, using the Neon transfection system (Thermo Fisher Scientific AG) according to the manufacturer’s instructions. Briefly, 10^6^ NHK cells were trypsinized, resuspended in 100 μl buffer R, added to 2 μg/ml of plasmid DNA and electroporated using the following parameters: pulse voltage 1100, pulse width 20, pulse number 2. Stably transfected cells were then selected with 1 μg/ml blasticidin and limiting dilution cloning was used to isolate a clone expressing high levels of TR. This clone was further transfected with pcDNA4/TO/hIL-38. Stable transfectants were selected with 100 μg/ml zeocin to obtain a population of NHK/38 cells containing both plasmids, which were maintained in 0.5 μg/ml blasticidin and 300 μg/ml zeocin. Alternatively, transfection with pcDNA4/TO/LacZ, followed by similar selection, yielded NHK/lacZ cells inducibly overexpressing E. Coli β-galactosidase, which were used as negative controls for immunofluorescence experiments. To induce IL-38 expression, NHK/38 cells were treated with Dox at 1 μg/ml for 24h. To promote differentiation of NHK/38 cells in monolayer (2D) cultures, medium was switched to DMEM (4.5 g/l glucose) containing 1.8 mM Ca^++^, supplemented with L-glutamine, streptomycin, penicillin and 10% FCS [32]. Primary human keratinocytes were grown in KBM-2 medium containing 0.06 mM Ca^++^. To induce differentiation in monolayer (2D) cultures, medium was additionally supplemented with 2 mM Ca^++^. For 3D differentiation of *in vitro* reconstructed human epidermis (RHE), 5x10^5^ primary juvenile keratinocytes were plated onto ThinCert cell culture inserts (Greiner Bio-One Vacuette Schweiz GmbH, St Gallen, Switzerland) and grown to confluency in CnT-Prime medium (CELLnTEC Advanced Cell Systems AG, Bern, Switzerland). The medium was then switched to CnT-Prime 3D Barrier medium and the cells cultured at the air-liquid interface. Human embryonic kidney (HEK) 293T cells were cultured in DMEM (4.5 g/l glucose) supplemented with L-glutamine, streptomycin, penicillin and 10% FCS. For transfection, HEK 293T cells were plated at a density of 2x10^5^ cells/ml and transfected 24 hours after seeding by calcium phosphate precipitation (Promega AG) with 0.5–1 μg/ml of pcDNA3.1/hIL-38 and/or pcDNA3.1/hDSTN (clone OHu30126C, Genescript Piscataway, NJ; GenBank accession n° NM_006870.3), or with empty pcDNA3.1(+) as a negative control.

### RNA extraction and RTqPCR

Total RNA was extracted from human epidermis and dermis using the mirVana RNA isolation kit (Thermo Fisher Scientific AG), from monolayer cultures of NHK and primary cells using the TRIzol® reagent (Life Technologies), and from RHE using the RNeasy Micro (Qiagen AG, Hombrechtikon, Switzerland) or the Quick-RNA Microprep (Zymo Research Corporation, Irvine, CA) kits. Total RNA was treated with RNAse free DNAse (Promega AG) and 250–500 ng were reverse transcribed using the SuperScript II Reverse transcriptase (Thermo Fisher Scientific AG). Gene expression levels were determined by quantitative PCR with a SYBR Green PCR master mix (Bio-Rad Laboratories AG, Cressier, Switzerland) and normalized to glucuronidase-β (*GUSB*) mRNA levels using a comparative method (2^-ΔCt^). Non-reverse-transcribed RNA samples and buffer were included as negative controls. The primer sequences (Eurofins, Ebersberg, Germany) used to detect IL-38 (*IL1F10*), keratin 10 (*KRT10*), involucrin (*IVL*), filaggrin (*FLG*) and *GUSB* are shown in Table 1.

**Table 1:** Primers used for qPCR.

Gene	Accession number	Primer sequence	Amplicon (bp)
*IL1F10*	NM_173161.3	Fwd 5’-CCCCATGGCAAGATACTAC-3’ Rev 5’-CCTCTTCTGTCTCCACACAT-3’	215
*KRT10*	NM_000421.4	Fwd 5'-TCCCAACTGGCCTTGAAACA-3' Rev 5'-TGAGAGCTGCACACAGTAGC-3’	75
*IVL*	NM_005547.3	Fwd 5'-TTGCTTCCTGTAGAGCACCA-3' Rev 5'-TAGCGGACCCGAAATAAGTG-3’	146
*FLG*	NM_002016.1	Fwd 5'-AAGGTTCACATTTATTGCCAAA-3' Rev 5'-GGATTTGCCGAAATTCCTTT-3’	157
*GUSB*	NM_000181.4	Fwd 5’-CCACCAGGGACCATCCAAT-3’ Rev 5’-AGTCAAAATATGTGTTCTGGACAAAGTAA-3’	79

### ELISA

NHK/38 cells were seeded in 96-well plates a density of 7.5x10^4^ cells/well and incubated on the following day with or without 1 μg/ml Dox for 24h. Culture supernatants were collected and cells were lyzed in culture medium containing 0.1% Triton X-100. To assess IL-38 release by RHE, culture media were collected 24h after the last medium change and concentrated 25 fold using a Centricon Plus-20 column (Merck & Cie, Schaffhausen, Switzerland) with a molecular weight cut-off of 5 kDa for cytokine detection by ELISA. RHE were lyzed by repeated freeze-thawing in 100 μl TNN buffer (50 mM Tris-HCl, pH 7.5, 150 mM NaCl, 1% Nonidet P-40) supplemented with a protease inhibitor cocktail (Sigma-Aldrich Chemie GmbH). Samples were cleared by centrifugation and total protein content was assessed with the DC protein assay kit (Bio-Rad Laboratories AG). IL-38 levels were determined using a human IL-38 DuoSet ELISA Development System (DY9110-05, R&D Systems).

### Histology and immunohistochemistry

RHEs were fixed in 4% formaldehyde and embedded in paraffin. For evaluation of the morphology, 5 μm sections were stained with hematoxylin (Merck & Cie) and eosin (Sigma-Aldrich Chemie GmbH). For immunohistochemistry, antigen retrieval was performed in 10 mM citrate buffer pH 6.0 for 30 min at 95°C. Sections were stained using the R.T.U. Vectastain Kit with ImmPACT AMEC Red Substrate (Vector Laboratories, Burlingame, CA, USA) according to the manufacturer’s protocol. The following antibodies were used: rabbit monoclonal anti-Ki67 (clone SP6, Thermo Fisher Scientific AG, 1/200), rabbit polyclonal anti-involucrin (Abcam, Cambridge, U.K., 1/1000), mouse monoclonal anti-filaggrin (clone FLG/1561, Abcam, 1/600) and rabbit monoclonal anti-keratin 10 (clone EP1607IHCY, Abcam, 1/5000). Sections were counterstained with haematoxylin solution, mounted with Glycerol Mounting Medium (Dako) and then images were taken using Zeiss Axioscan.Z1 (Carl Zeiss Microscopy) and analyzed with ZEN black software (Carl Zeiss Microscopy).

### Immunofluorescence

NHK/38 and NHK/lacZ cells were seeded in chamber slides at a density of 1.6x10^5^ cells/well and incubated on the following day with or without 1 μg/ml Dox for 24h. HEK 293T cells were seeded in chamber slides, transiently transfected with pcDNA3.1/hIL-38, pcDNA3.1/hDSTN, or empty pcDNA3.1(+) as a control, and used 24 to 48h after transfection. Cells were fixed (PBS, 2% paraformaldehyde), permeabilized (PBS, 0.1% Triton X-100) and washed (Dako wash buffer, Agilent Technologies AG), before staining with a polyclonal goat anti-IL38 antibody (AF2427, R&D Systems, Abingdon, UK, 1/1000), a monoclonal mouse anti-hIL-38 antibody (H127C, Thermo Fisher Scientific AG, 1/2000), a polyclonal rabbit anti-DSTN antibody (PA1-24937, Thermo Fisher Scientific AG, 1/200), a monoclonal mouse anti-GAPDH antibody (MAB374, clone 6C5, Sigma-Aldrich Chemie GmbH, 1/200) and/or FITC-labeled phalloidin (P5282, Sigma-Aldrich Chemie GmbH, 25 μg/ml) in antibody diluent (Dako, Agilent Technologies AG) overnight at 4°C. For RHE and human skin biopsies, tissue sections were deparaffinized and antigens retrieved by microwave heating in 10 mM citrate buffer, pH 6. Slides were permeabilized, washed and incubated with a monoclonal mouse anti-hIL-38 antibody (H127C, Thermo Fisher Scientific AG, 1/2000) and/or a polyclonal rabbit anti-DSTN antibody (PA1-24937, Thermo Fisher Scientific AG, 1/200) in antibody diluent overnight at 4°C. Subsequently, primary antibodies were detected with rhodamine-labeled donkey anti-goat IgG (705-025-003, Jackson Immuno Research Europe Ltd, Ely, UK, 1/200), Alexa Fluor 594-labeled goat anti-mouse IgG2b (115-585-207, Jackson Immuno Research Europe Ltd, 1/200), Alexa Fluor 555-labeled goat anti-mouse IgG (A21422, Thermo Fisher Scientific AG, 1/800), Alexa Fluor 594-labeled donkey anti-rabbit IgG (A21422, Thermo Fisher Scientific AG, 1/1000), and/or Alexa Fluor 488-labeled donkey anti-rabbit IgG (711-545-152, Jackson Immuno Research Europe Ltd, 1/200) secondary antibodies, as appropriate, and slides were stained with 4’,6-diamidino-2-phenylindole (DAPI; D3571,Thermo Fisher Scientific AG, 1/2000 in PBS) and mounted in FluoreGuard medium (ScyTek Laboratories, Inc., Logan, UT). To assess staining specificity, negative controls were performed with isotype-matched control antibodies or in absence of primary antibodies. In addition, the specificity of IL-38 detection was assessed in monolayer cultures by using control cells lacking IL-38 expression and in RHE by preadsorbing the anti-IL-38 antibody with a 20-fold molar excess of recombinant human IL-38 (AG-40A-0191, Adipogen AG) before use. Slides were imaged with a LSM700 confocal microscope (Carl Zeiss Microscopy, Feldbach, Switzerland) and the ZEN black software (Carl Zeiss Microscopy). Co-localization was analyzed on single confocal sections using the Coloc module of Imaris 9.3.1 (Bitplane AG, Zurich, Switzerland) with global manual thresholds set according to the fluorescence signal intensities of isotype-matched control antibody-stained samples. Results were represented using the co-localization channel tool of the software. Thresholded Manders’ split co-localization coefficients were computed in 1–5 random microscopic fields for each sample.

### Yeast 2 hybrid

An ULTImate yeast 2 hybrid (Y2H) screen was performed by Hybrigenics Services (Paris, France), using amino acids (aa) 30–152 of human IL-38, C-terminally fused to the LexA DNA-binding domain (DBD), as a bait. Indeed, IL-38 was suggested to undergo N-terminal processing by as yet unidentified protease(s) [8], which might in principle be conserved in yeast. We thus used an N-terminally truncated form of IL-38, starting downstream of the proposed cleavage sites, to avoid potential proteolytic processing between the DBD and the IL-38 portion of the bait. The N-LexA(DBD)-IL-38(aa30-152)-C fusion construct was cloned into the pB27 vector and used to screen a human reconstituted skin (EpiSkin, Lyon, France)_RP2 prey library (complexity ≥10^7^ primary clones) at a 10-fold coverage. A high stringency 3-amino-1,2,4-triazole (3-AT) concentration (100.0 mM) was used to overcome auto-activation of the bait fusion.

### Immunoprecipitation and Western blotting

HEK 293T were seeded in 10-cm petri dishes and transiently transfected with pcDNA3.1/hIL-38 and pcDNA3.1/hDSTN. Forty-eight hours after transfection, cellular proteins were crosslinked with dithiobis(succinimidylpropionate) (DSP, Thermo Fisher Scientific AG), extracted in lysis buffer (20 mM Tris/HCl, pH 8, 137 mM NaCl, 1% Nonidet P-40, 2 mM EDTA) supplemented with a protease inhibitor cocktail for 30 min on ice and cleared by centrifugation. 4x10^6^ cell equivalents were immunoprecipitated with a polyclonal rabbit anti-DSTN antibody (PA1-24937, Thermo Fisher Scientific AG, 4 μg) or with normal rabbit IgG (10500C, Thermo Fisher Scientific AG, 4 μg) as a negative control. Alternatively, immunoprecipitation (IP) was performed with a polyclonal goat anti-IL38 antibody (AF2427, R&D Systems, 4 μg) or with normal goat IgG (005-000-003, Jackson Immuno Research Europe Ltd 4 μg) as a negative control. NHK/38 cells were seeded in 6-well plates at a density of 8x10^5^ cells/well and incubated on the following day with or without 1 μg/ml Dox for 24h. Cells were then lyzed by freeze-thawing in TN buffer supplemented with protease inhibitors. Immunoprecipitated proteins and total cell lysates (3x10^4^ cell equivalents for HEK 293T cells, 8x10^4^ cell equivalents for NHK/38 cells) were separated electrophoretically on a 4–12% gradient SDS-PAGE (NuPAGE; Thermo Fisher Scientific AG) and blotted onto PVDF membranes. The membranes were blocked with 5% horse serum in TBS, 0.05% Triton X-100 (TBST) and probed with a biotinylated polyclonal goat anti-IL38 antibody (BAF2427, R&D Systems, 1/1000) or with a polyclonal rabbit anti-DSTN antibody (PA1-24937, Thermo Fisher Scientific AG, 1/5000) in TBST. Immunoreactive bands were detected using streptavidin HRP (BD Biosciences, San Jose, CA, 1/3000) or an HRP-labeled anti-rabbit IgG secondary antibody (Bio-Rad Laboratories AG, 1/10’000) and Radiance Plus chemiluminescent detection (Azure Biosystems, Dublin, CA) on a LAS4000 imager (Fujifilm Life Science, Düsseldorf, Germany). After stripping in Re-Blot Plus Strong antibody stripping solution (Merck & Cie) for 20 min at RT and blocking with TBST, 5% horse serum, the membranes were reprobed with the anti-DSTN antibody, the anti-IL38 antibody or a monoclonal mouse anti-GAPDH antibody (MAB374, clone 6C5, Sigma-Aldrich Chemie GmbH, 1/2000) and appropriate HRP-labeled secondary reagents for ECL detection.

### Proximity ligation assay

NHK/38 cells were seeded in chamber slides at a density of 1.6x10^5^ cells/ and incubated on the following day with or without 1 μg/ml Dox for 24h. Cells were fixed (PBS, 2% paraformaldehyde, then methanol), permeabilized (PBS, 0.1% Triton X-100) and washed (Dako wash buffer, Agilent Technologies AG), before co-incubation with a polyclonal goat anti-IL38 antibody (AF2427, R&D Systems, 1/1000) and a polyclonal rabbit anti-DSTN antibody (PA1-24937, Thermo Fisher Scientific AG, 1/200) in antibody diluent (Dako, Agilent Technologies AG). Proximity ligation assay (PLA) probes (DuoLink, Sigma-Aldrich Chemie GmbH, Buchs, Switzerland), diluted in antibody diluent, were then added to the slides, ligated, amplified and washed, according to the manufacturer’s instructions. Slides were mounted using the DuoLink in situ DAPI containing mounting medium. Negative controls were performed by incubation of 24h Dox-treated and untreated NHK/38 cells with only one of the primary antibodies or with antibody diluent alone. Slides were imaged with a LSM700 confocal microscope (Carl Zeiss Microscopy) and the ZEN black software (Carl Zeiss Microscopy). PLA results were quantified by manually counting the number of positive cells containing at least one PLA signal, normalized to the total number of cells per field, and the mean number of PLA spots per positive cell in 2–4 random microscopic fields for each sample.

### Statistical analysis

Data were analyzed with Prism version 8 (Graphpad Software, La Jolla, USA) using a one-way ANOVA followed by Tukey’s multiple comparisons test or an unpaired Student’s t test, as indicated. Values are expressed as mean ± SEM. Statistical significance was defined at a *p*-value < 0.05.

## Results

**Fig 1:**
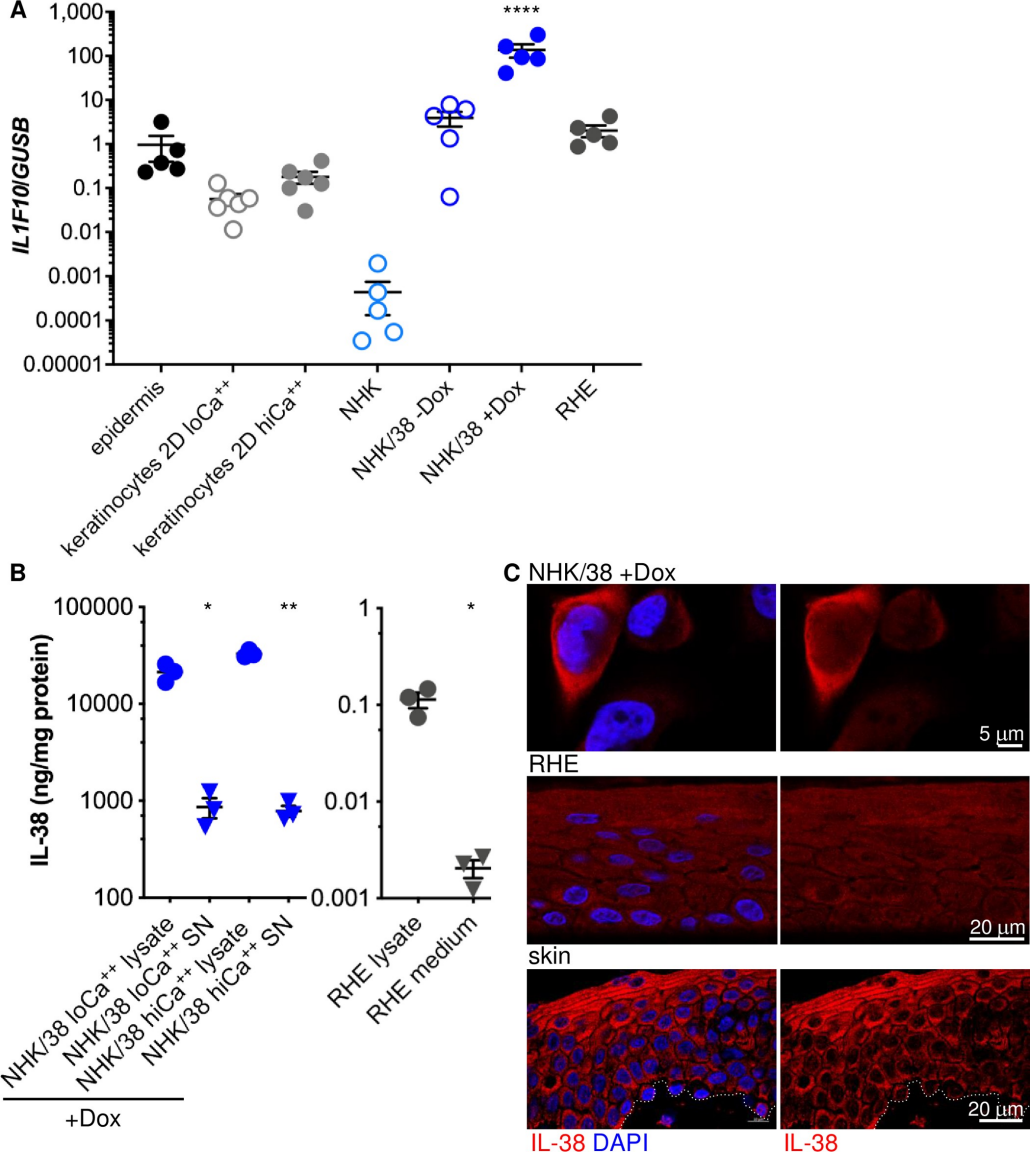
IL-38 expression and localization in human keratinocytes. **A**. *IL-38 (IL1F10)* mRNA expression, quantified by RTqPCR, is shown for normal human epidermis, primary human keratinocytes cultured in monolayers (2D) in the presence of low (lo; 0.06 mM) or high (hi; 2 mM) Ca^++^, parental NHK cells, NHK/38 cells without or with 24h Dox treatment, and RHE. *IL-38 (IL1F10)* mRNA levels are expressed relative to *GUSB*. Results are shown as individual values and mean ± SEM for n = 5–6 different donors (epidermis and primary cells) or independent cultures (NHK cells). ****p<0.0001 vs. epidermis, primary 2D or RHE cultures, NHK and NHK/38 –Dox cells, as assessed by one-way ANOVA, followed by Tukey’s multiple comparisons test. **B.** IL-38 protein levels, assessed by ELISA, are shown in cell lysates and culture supernatants (SN) of Dox-treated NHK/38 cells kept in proliferation medium (loCa^++^) or switched to 1.8 mM Ca^++^ containing (hiCa^++^) differentiation medium for 3 days (left panel), as well as in RHE and in their 24h-conditioned media (right panel). Results are expressed as ng IL-38 per mg of protein in the cell layer(s). Data are shown as individual values and mean ± SEM for n = 3 cultures (NHK/38 cells) or 3 different donors (RHE) respectively. *p<0.05, **p<0.001 vs. lysate, as assessed by paired Student’s t test. **C.** IL-38 protein expression in 24h Dox-treated NHK/38 cells (upper panels), RHE (middle panels) and normal human skin (lower panels) was assessed by IF (red staining; all panels). Nuclei were labeled with DAPI (blue staining; left panels). Results are representative of 5 independent experiments (NHK/38 cells) or 3 different donors (RHE and skin). Dotted lines outline the epidermal-dermal border. Original magnification 63x.

*IL-38 (IL1F10)* mRNA was constitutively expressed in normal human epidermis (IL-38/GUSB: 0.957 ± 0.563) (Fig 1A). In contrast, *IL-38* levels in the dermis were low (IL-38/GUSB: 0.009 ± 0.008), in agreement with previous studies suggesting that keratinocytes are the main source of IL-38 in the skin [4, 30, 33]. The mean *IL-38* expression level was reduced more than 15 fold when primary human keratinocytes were isolated from epidermal sheets and grown in 2D culture, and only partially restored when the cells were cultured in presence of a high Ca^++^ concentration (2mM) to favor differentiation (Fig 1A). We thus set up a doxycycline (Dox)-inducible IL-38 overexpression system using an immortalized normal human keratinocyte (NHK) cell line [32] to investigate the localization and release of this cytokine in monolayer cultures. Parental NHK cells expressed very low amounts of endogenous *IL-38* mRNA (Fig 1A) and IL-38 protein levels were below the detection limit of a commercially available ELISA (S1A Fig). IL-38 overexpressing NHK (NHK/38) cells already produced *IL-38* mRNA (Fig 1A) and low levels of IL-38 protein (S1 Fig) in the absence of Dox, reflecting leaky expression from the inducible system at baseline. Treatment with Dox for 24h further enhanced IL-38 mRNA (Fig 1A) and protein (S1A–S1C Fig) expression in NHK/38 cells.

In parallel, we used primary human keratinocytes in a 3D air-lift culture model to generate *in vitro* reconstructed human epidermis (RHE). This 3D culture system allowed for efficient induction of keratinocyte differentiation, as indicated by enhanced mRNA expression of differentiation markers, such as keratin 10 (KRT10), involucrin (INV) and filaggrin (FLG), as compared to cells in 2D culture, in low or high Ca^++^-containing media (S2A Fig). After 10 days of culture at the air-liquid interface, the cells formed a morphologically well-defined epidermis-like structure including a basal, spinous, granular and cornified layer (S2B Fig). Immunohistochemistry confirmed the presence of proliferating Ki-67 positive cells in the basal layer and sequential expression of the differentiation markers KRT10, IVL and FIL in the upper layers of the epithelium (S2B Fig). Consistent with previously published data [30], the culture of primary keratinocytes in RHE restored endogenous IL-38 mRNA and protein expression (Fig 1).

We then used Dox-treated NHK/38 cells to investigate extracellular release of IL-38 from monolayer cultures. In parallel, we examined release of endogenous IL-38 from primary human keratinocytes in RHE. In both instances, IL-38 was mainly (>95%) cell-associated, and only a minor fraction of the protein was recovered in culture media (Fig 1B). We also examined the intracellular localization of the protein by immunofluorescence (IF). Confocal analysis indicated that cell-associated IL-38 was preferentially, although not exclusively, cytoplasmic in NHK/38 cells and in RHE (Fig 1C). In agreement with published data [9], IL-38 staining was also detected in the epidermis of normal human skin, where the cytokine was predominantly cytoplasmic and essentially excluded from keratinocyte nuclei (Fig 1C). The specificity of the anti-IL-38 staining in monolayer cultures, was validated by using NHK/38 cells without Dox-treatment, as well as cells lacking IL-38 expression as negative controls (S1C and S3 Figs). The AF2427 polyclonal and the H127C monoclonal anti-IL-38 antibodies used in this study performed similarly and produced comparable staining patterns in cell monolayers (S3 Fig). For detection of IL-38 in RHE and normal human skin, we favored the use of the H127C monoclonal antibody, which has a better signal-to-noise ratio on paraffin sections, and the specificity of IL-38 detection was assessed by using isotype controls and pre-adsorption of the anti-IL-38 antibody with an excess of recombinant IL-38 protein (S4 Fig).

As information concerning the biology, regulation and mechanism(s) of action of IL-38 is still scarce, we initiated a Y2H screen to identify IL-38-interacting proteins in the epidermis. Human IL-38 (aa 30–152) was used as a bait to probe a reconstructed human epidermis library, revealing high confidence interactions between IL-38 and six potential binding partners (Table 2).

**Table 2:** Y2H preys.

Gene Name	GenBank ID (NCBI)
Destrin—DSTN	http://www.ncbi.nlm.nih.gov/nuccore/ 58530847
DNA-binding protein inhibitor—ID1	http://www.ncbi.nlm.nih.gov/nuccore/ 341865544
peroxiredoxin 1—PRDX1	http://www.ncbi.nlm.nih.gov/nuccore/ 320461710
peroxiredoxin 2—PRDX2	http://www.ncbi.nlm.nih.gov/nuccore/ 460417277
ring finger protein 39—RNF39	http://www.ncbi.nlm.nih.gov/nuccore/ 297206762
thrombospondin 2—THBS2	http://www.ncbi.nlm.nih.gov/nuccore/ 538918410

List of preys identified in an ULTImate Y2H SCREEN by using a human IL-38 (aa 30–152) bait fragment to screen the human reconstituted skin_RP2 prey library. Vector: pB27 N-LexA-bait-C fusion. Processed clones: 255. Analyzed interactions: 144 millions. 3AT concentration: 100.0 mM. Results were ranked into 4 categories from A (highest confidence rank) to D. Only A-ranked preys are listed.

**Fig 2:**
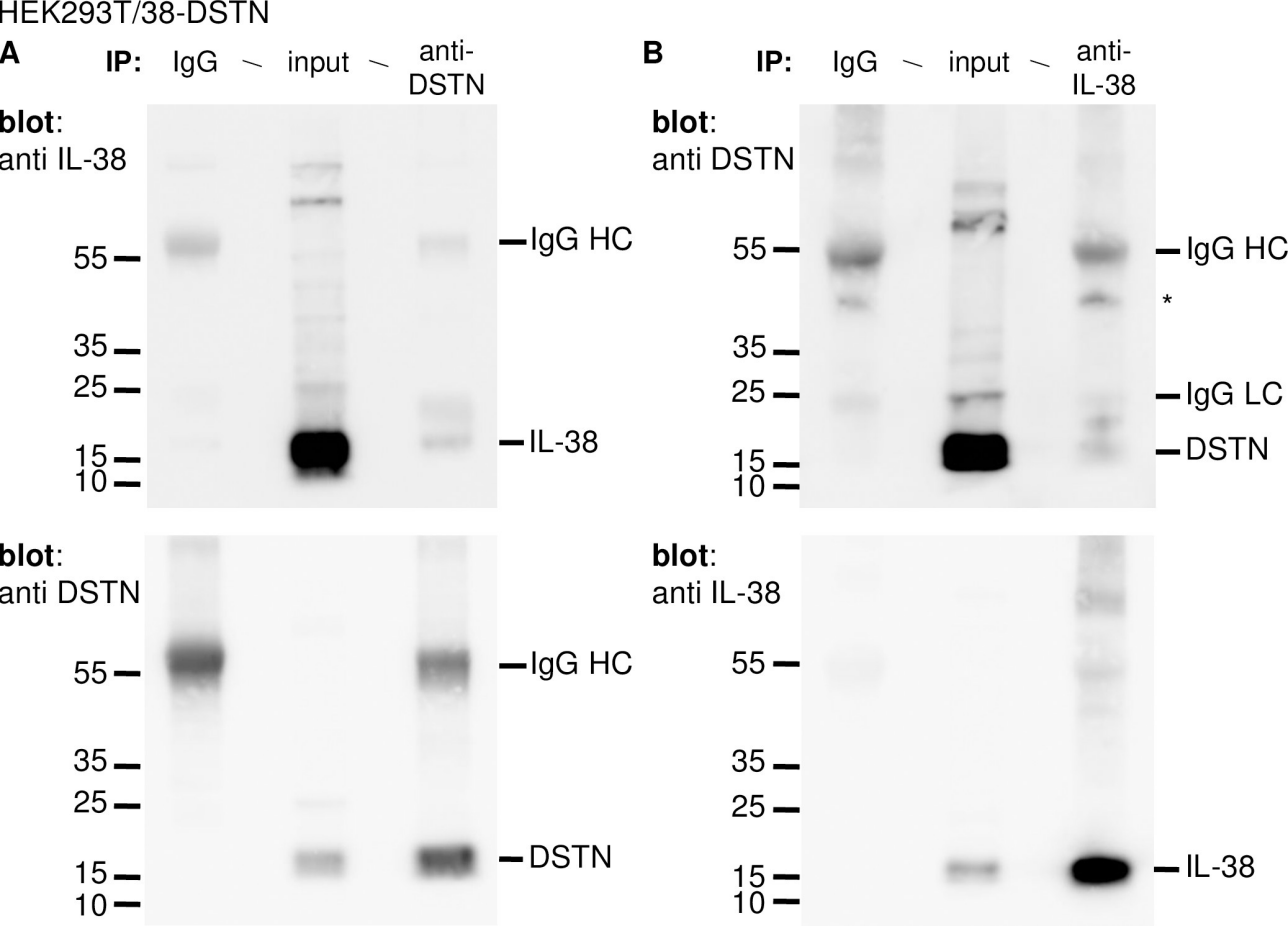
Interaction of IL-38 and DSTN in HEK293T cells. Association of IL-38 and DSTN was examined by co-IP in HEK293T cells co-transfected with pcDNA3.1/hIL-38 and pcDNA3.1/hDSTN. **A**. Cells were treated with DSP, lyzed and immunoprecipitated with a polyclonal rabbit anti-DSTN antibody (right lane), or with normal rabbit IgG (left lane) as a negative control. Whole-cell lysates (input; middle lane) and immunoprecipitated proteins were fractionated by SDS-PAGE and IL-38 was detected by Western blotting (upper panel). The membrane was then stripped and reprobed with the anti-DSTN antibody (lower panel). **B.** Cells were treated with DSP, lyzed and immunoprecipitated with a polyclonal goat anti-IL-38 antibody (right lane), or with normal goat IgG (left lane) as a negative control. Whole-cell lysates (input; middle lane) and immunoprecipitated proteins were fractionated by SDS-PAGE and DSTN was detected by Western blotting (upper panel). The membrane was then stripped and reprobed with the anti-IL-38 antibody (lower panel). The positions of IL-38 and DSTN, as well as of the heavy (HC) and light (LC) chains of the IgGs used for IP are indicated on the right of the blots. *indicates a non-specific band present also in the negative control IP. Molecular mass markers are indicated on the left of each blot.

The highest number of interacting clones was obtained for destrin/actin-depolymerizing factor (DSTN). Indeed, 42 prey fragments sharing a sequence corresponding to aa 43–148 of DSTN were isolated in this experiment. Interaction between IL-38 and DSTN was confirmed by co-immunoprecipitation of both interaction partners overexpressed in HEK 293T cells (Fig 2).

**Fig 3:**
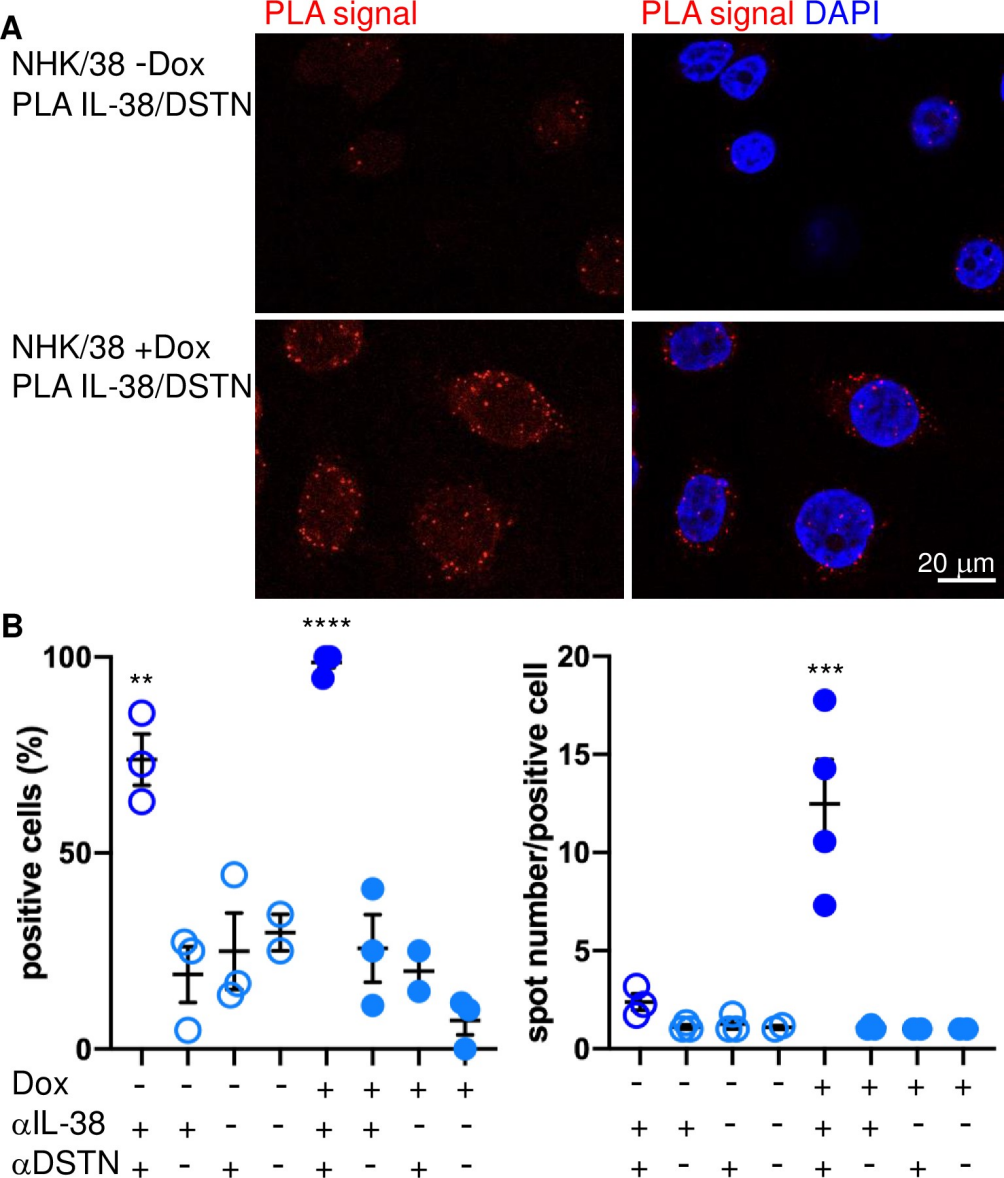
Interaction of IL-38 and DSTN in NHK/38 cells. **A.** A proximity ligation assay (PLA) was used to probe co-localization of IL-38 and DSTN in NHK/38 cells without (upper panels) or with (lower panels) 24h Dox treatment. After addition of PLA probes and signal amplification, PLA signals, indicating close proximity of the two target proteins, were visualized as discrete red spots (all panels). Nuclei were labeled with DAPI (blue staining, right panels). Original magnification 63x. **B.** PLA results were quantified in NHK/38 cells without (-) or with (+) 24h Dox treatment by assessing the percentage of positive cells containing at least one PLA signal and the mean number of PLA spots per positive cell. Negative controls were performed by incubating untreated and Dox-treated NHK/38 cells with the anti-DSTN (αDSTN) antibody alone, the anti-IL-38 antibody (αIL-38) alone or antibody diluent only and quantified in parallel. Results are shown as individual values and mean ± SEM. **p<0.01, ***p<0.001, ****p<0.0001 vs. negative controls incubated in absence of one or both antibodies, as assessed by one-way ANOVA, followed by Tukey’s multiple comparisons test.

Molecular proximity between IL-38 and endogenous DSTN was further confirmed in NHK/38 cells using a proximity ligation assay (PLA) (Fig 3; see S5 Fig for additional controls). Multiple PLA signals, indicating spatial co-occurrence of IL-38 and DSTN, were observed in 99% of the Dox-treated NHK/38 cells.

**Fig 4:**
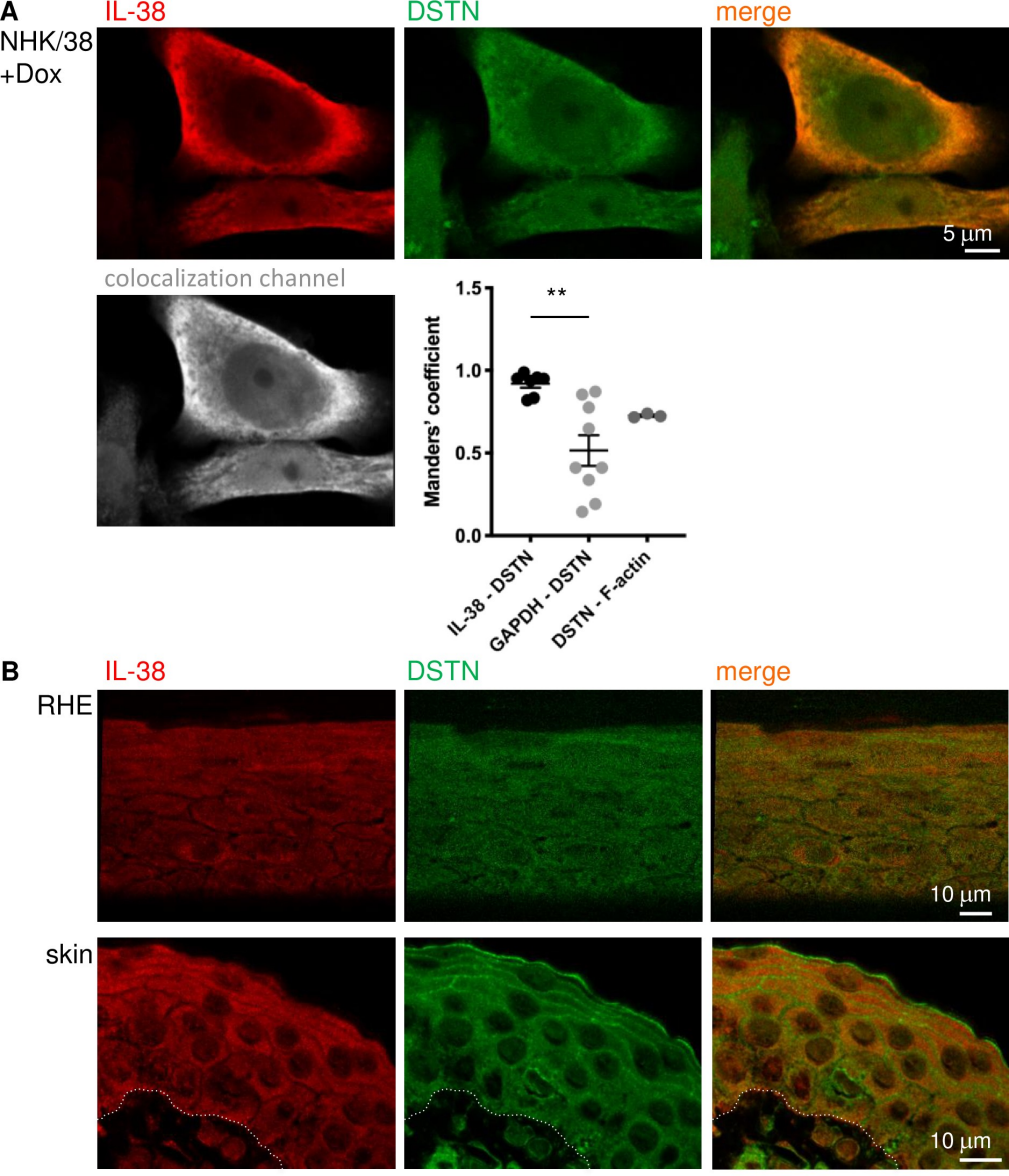
Co-localization of IL-38 and DSTN in human keratinocytes. **A.** Localization of IL-38 (red staining; upper left and right panels) and DSTN (green staining; upper middle and right panels) was examined by confocal IF microscopy in 24h Dox-treated NHK/38 cells. Overlap between the red and green fluorescence signals is visible in yellow in the merged image (upper right panel) and co-localization between IL-38 and DSTN, as assessed using the Imaris Coloc module, is illustrated in white in the co-localization channel (lower left panel). Thresholded Manders’ coefficients for co-localization of IL-38 with DSTN, GAPDH with DSTN and DSTN with F-actin are shown as individual values and mean ± SEM for n = 3–9 independent microscopic fields. **p<0.01 IL-38/DSTN vs. GAPDH/DSTN. **B.** Localization of IL-38 (red staining; left and right panels) and DSTN (green staining; middle and right panels) is shown for RHE (upper row) and normal human skin (lower row). Partial spatial co-occurrence of IL-38 and DSTN is visible in yellow in the merged images (right panels). Results are representative of 3 cultures (NHK/38 cells), or 3 (RHE) or 2 (skin) different donors respectively. Dotted lines outline the epidermal-dermal border. Original magnification 63x.

**Fig 5:**
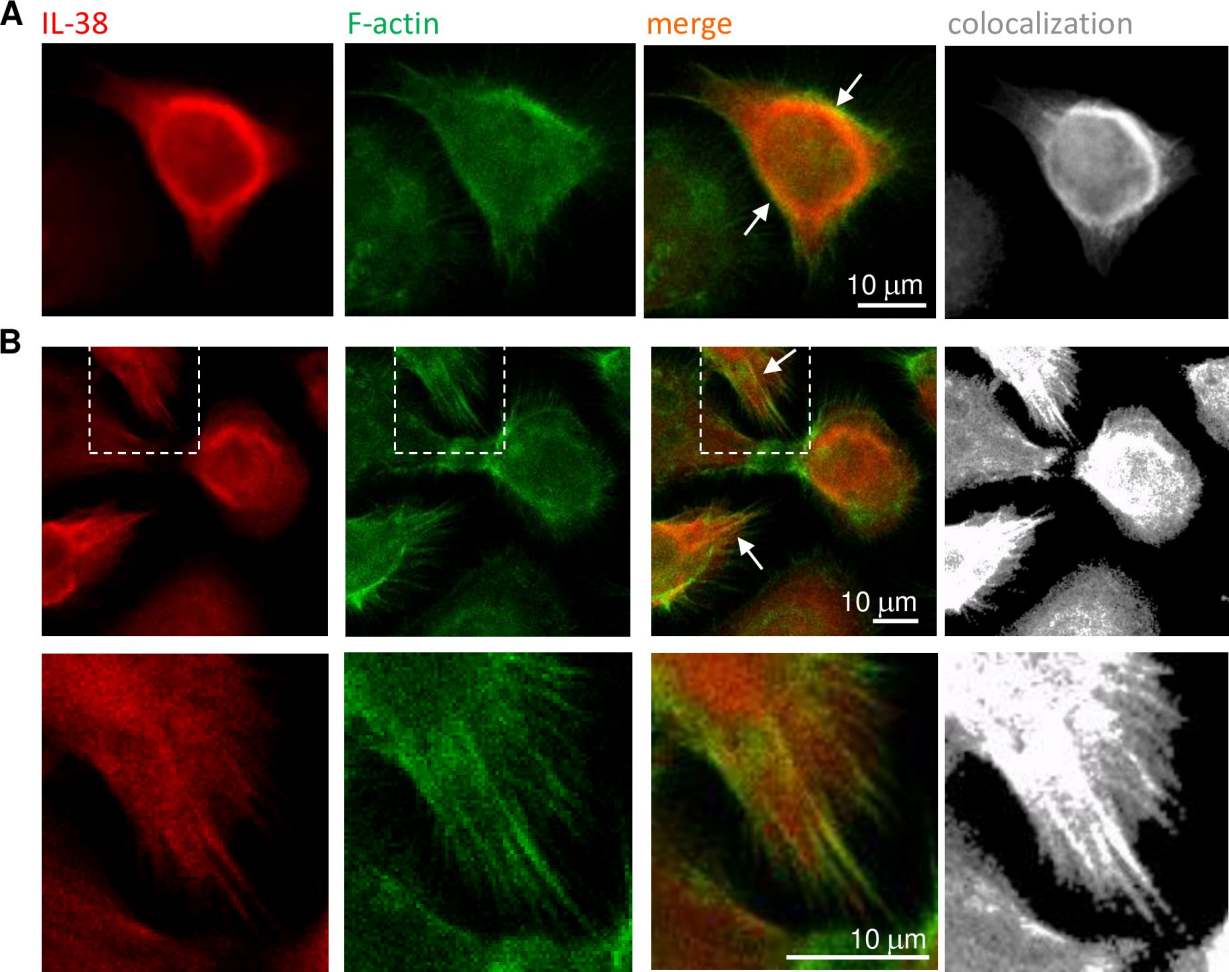
Co-localization of IL-38 and F-actin in NHK/38 cells. Localization of IL-38 (red staining; first and third panels in each row) and F-actin (green staining; second and third panels in each row) was examined by confocal IF microscopy in 24h Dox-treated NHK/38 cells. Spatial co-occurrence of IL-38 and F-actin is visible in yellow in the merged images (third panel in each row) and co-localization between IL-38 and F-actin, as assessed using the Imaris Coloc module, is illustrated in white (co-localization channel; last panel in each row). **A.** Co-localization of IL-38 and F-actin was observed in particular on the inner face of the cortical F-actin network (arrows). **B.** Co-localization of IL-38 and F-actin was also observed in filopodia (arrows, upper panels). A smaller ROI is magnified and shown in the lower panels. Results are representative of 2 experiments. Original magnification 63x.

The specificity of DSTN detection by IF was validated in cultured cells by DSTN overexpression and on paraffin sections by using isotype-matched control antibodies and staining with secondary antibodies only (S6 Fig). By confocal microscopy, we then confirmed partial co-localization of IL-38 and DSTN in NHK/38 cells in 2D culture, which was most striking in peripheral cytoplasmic regions (Fig 4A). We similarly examined co-localization of DSTN and F-actin, a known DSTN-interaction partner, which we used as a positive control, and of GAPDH and DSTN, as an example of two proteins not necessarily expected to co-localize (S7 Fig). IL-38 co-localized significantly more with DSTN than GAPDH, as indicated by the respective Manders’ co-localization coefficients for these proteins, while IL-38/DSTN and DSTN/F-actin co-localization was comparable (Fig 4A). Endogenous IL-38 and DSTN were also co-expressed throughout the epidermis in RHE and in normal human skin (Fig 4B). Finally, in NHK/38 2D cultures, IL-38 partially co-localized with F-actin (thresholded Manders’ coefficient: IL-38/F-actin 0.803 ± 0.053, vs. DSTN/F-actin 0.727 ± 0.007, ns; for n = 3 independent microscopic fields in one experiment), in particular on the inner face of the cortical actin network and in filopodia (Fig 5).

## Discussion

In the present study, we investigated the subcellular localization of the IL-38 protein in human keratinocytes using IL-38 overexpressing NHK/38 cells and RHE. Consistent with its lack of a signal peptide for conventional secretion, IL-38 was mainly cell-associated in cultured keratinocytes, both in monolayers and in RHE. It thus appears that neither proliferating nor differentiated keratinocytes spontaneously release major amounts of IL-38. Most IL-1 family cytokines lack signal sequences to direct them towards the classical secretory pathway and many are released into the extracellular space upon cell stress or cell death [34]. Pyroptosis and necroptosis, for instance, are frequently associated with the release of IL-1β and IL-18 [35]. Other IL-1 family members, such as IL-1α and IL-33, are typically released during various forms of lytic cell death, thereby acting as alarmins to signal cell damage to the immune system [36]. Finally, a previous study also reported IL-38 release during apoptosis [8]. It is thus conceivable that, in particular situations, cell stress, activation and/or death, might cause IL-38 to be either passively released, or even actively secreted. Indeed the related IL-1 family antagonist IL-1Ra, which is also constitutively produced as an intracellular protein in keratinocytes, was shown to be released in response to mechanical stress or cytokine treatment [37–39]. In addition, the activity of many IL-1 family cytokines is enhanced by N-terminal processing [40]. This led to the suggestion that mature bioactive IL-38 might correspond to an N-terminally truncated form of the protein [8]. However, specific cleavage site(s) and protease(s) involved still remain to be characterized [8, 41–43].

Cell-associated IL-38 was predominantly cytoplasmic in cultured keratinocytes and in normal human epidermis. A Y2H screen revealed several potential IL-38-interacting molecules. Interestingly, except for thrombospondin 2, all recovered clones encoded intracellular proteins. High confidence interaction was observed in particular with the cytoplasmic protein DSTN. This interaction was confirmed by co-immunoprecipitation of IL-38 and DSTN co-expressed in HEK 293T cells. Molecular proximity of IL-38 with endogenous DSTN was further validated by PLA in NHK/38 keratinocytes, where partial co-localization of IL-38 and DSTN was also observed by IF. DSTN, together with the related protein cofilin-1, was previously shown to be essential for normal keratinocyte differentiation and skin homeostasis, through its inhibitory effects on actin polymerization and stress fiber formation [44]. Consistently, DSTN expression was detected in keratinocytes of all epidermal layers in RHE and in normal human skin, where it partially co-localized with IL-38, suggesting that the two binding partners may also interact *in vivo*. The functional consequences of this interaction are currently unclear. Intracellular binding partners could be involved in the regulation of IL-38 protein levels or function by affecting its localization, stability, maturation, and/or release. Alternatively, as suggested for other IL-1 family members [45–48], intracellular IL-38 binding proteins might represent *bona fide* interaction partners for intracellular functions of the cytokine. Conversely, interaction with IL-38 might modulate DSTN function and thereby actin filament dynamics and cell homeostasis. Interestingly in this context, loss of DSTN function was previously shown to induce an inflammatory phenotype in the corneal epithelium [49].

Finally, IL-38 partially co-localized with F-actin in NHK/38 cells, in particular on the inner face of the cortical actin network. Furthermore, as previously described [50], isolated keratinocytes in culture extended numerous filopodia, lamellipodia and ruffles and IL-38 also co-localized with actin filaments in filopodia. It is tempting to speculate that the proximity of IL-38 with the edge of the cells could facilitate the release of this cytokine in stress situations or upon cell activation. In fact, despite the undisputed importance of the IL-1 and IL-36 systems in skin inflammatory responses, the mechanisms regulating the unconventional leaderless secretion and/or the passive release of the two constitutively expressed antagonists IL-1Ra and IL-36Ra by keratinocytes are still unclear. It will thus be informative to explore whether intracellular localization and/or interaction partners are shared between IL-38 and other IL-1 family members.

In conclusion, at steady state, IL-38 is predominantly found in the cytoplasm of human keratinocytes, where it interacts with DSTN. We observed partial co-localization of these two proteins, as well as of IL-38 and F-actin. The functional relevance of this interaction remains to be investigated.

## Supporting information

S1 Fig IL-38 expression in NHK/38 cells. **A.** IL-38 protein levels in NHK/38 cell lysates after 24h incubation without or with Dox were assessed by ELISA. Results are expressed as ng IL-38 per mg of cellular protein. Data are shown as individual values and mean ± SEM for triplicate wells in one experiment representative of 4 independent cultures. *p<0.05 vs. NHK and NHK/38 -Dox cells, as assessed by one-way ANOVA, followed by Tukey’s multiple comparisons test. **B.** IL-38 protein expression in NHK/38 cells without or with 24h Dox treatment was assessed by Western blotting (upper panel). The membrane was then stripped and reprobed with an anti-GAPDH antibody as a loading control (lower panel). Results are representative of 2 experiments. **C.** IL-38 protein expression in NHK/38 cells without (left panels) or with (right panels) 24h Dox treatment was assessed by IF (red staining; all panels). Nuclei were labeled with DAPI (blue staining; upper panels). Results are representative of 5 independent experiments. Original magnification 63x.
(PPTX)

S2 Fig Epidermal differentiation of primary human keratinocytes in RHE. **A.** *KRT10* (left panel), *IVL* (middle panel) and *FLG* (right panel) mRNA levels were assessed by RT-qPCR in primary human keratinocytes cultured in monolayers (2D) in presence of low (lo; 0.06mM) or high (hi; 2mM) Ca^++^, or in RHE. Transcript levels are expressed relative to *GUSB*. Results are shown as individual values and mean ± SEM for 6 (2D cultures) or 4 (RHE) different donors. *p<0.05, ****p>0.0001 vs. 2D cultures, as assessed by one-way ANOVA, followed by Tukey’s multiple comparisons test. **B.** The structure of the RHE was examined by HE staining (upper left panel). After 10 days of culture at the air-liquid interface, the cells formed a morphologically well-defined epidermis-like structure with (from bottom to top) basal (B), spinous (S), granular (G) and cornified (C) layers similar to *in vivo* skin. Protein expression of keratinocyte proliferation (Ki67; brown staining, upper right panel) and differentiation (KRT10, IVL, FLG; brown staining, lower panels) markers was assessed by IHC. Original magnification 10x.
(PPTX)

S3 Fig Specificity of IL-38 detection by IF in cell monolayers. IL-38 was detected by IF in HEK 293T cells transfected with pcDNA3.1/hIL-38 (red staining, overexpressed IL-38; upper panels) or with empty pcDNA3.1 as a negative control (lower panels) using the AF2427 polyclonal goat anti-IL-38 antibody (**A**) or the H127C monoclonal mouse anti-IL-38 antibody (**B**). IL-38 was detected by IF in 24h Dox-treated NHK/38 cells (red staining, overexpressed IL-38; upper panels) or NHK/lacZ cells used as a negative control (lower panels) using the AF2427 polyclonal goat anti-IL-38 antibody (**C**) or the H127C monoclonal mouse anti-IL-38 antibody (**D**). Nuclei were labeled with DAPI (blue staining; left panels). Original magnification 40x.
(PPTX)

S4 Fig Specificity of IL-38 detection by IF in RHE and skin. **A.** IL-38 was detected by IF in RHE using a monoclonal mouse anti-IL-38 antibody (red staining; upper panels) or normal mouse IgG as a negative control (lower panels). Nuclei were labeled with DAPI (blue staining; left panels). Results are representative of 5 independent experiments. Original magnification 63x. **B.** IL-38 protein expression in RHE was examined by IF using a monoclonal mouse anti-IL-38 antibody (red staining; upper panels) or the same antibody pre-adsorbed with recombinant human IL-38 (lower panels). Nuclei were labeled with DAPI (blue staining; left panels). Results are representative of 2 independent experiments. Original magnification 63x. **C.** IL-38 protein expression in normal human skin was assessed by IF using a monoclonal mouse anti-IL-38 antibody (red staining; upper panels) or normal mouse IgG as a negative control (lower panels). Nuclei were labeled with DAPI (blue staining; left panels). Results are representative of 3 different donors. Dotted lines outline the epidermal-dermal border. Original magnification 40x.
(PPTX)

S5 Fig Specificity of the detection of IL-38-DSTN interactions by PLA. Negative controls for the PLA experiment were performed by incubation of 24h Dox-treated NHK/38 cells with the anti-DSTN antibody alone (upper panels), the anti-IL-38 antibody alone (middle panels) or antibody diluent only (lower panels). After addition of PLA probes and signal amplification, only minimal background staining was observed (red staining; all panels). Nuclei were labeled with DAPI (blue staining, right panels). Original magnification 63x.
(PPTX)

S6 Fig Specificity of DSTN detection by IF in cell monolayers, RHE and skin. **A.** DSTN was detected by IF in HEK 293T cells transfected with pcDNA3.1/hDSTN (green staining, overexpressed DSTN; upper panels) or empty pcDNA3.1 (green staining, endogenous DSTN; middle panels) using a polyclonal rabbit anti-DSTN antibody. Staining with normal rabbit IgG, used as a negative control, is shown for HEK293T cells transfected with pcDNA3.1/hDSTN (lower panels). Nuclei were labeled with DAPI (blue staining; left panels). Original magnification 20x. **B.** DSTN was detected by IF in RHE using a polyclonal rabbit anti-DSTN antibody (green staining; upper panels). Detection with the labeled secondary anti-rabbit antibody alone is shown as a negative control (lower panels). Nuclei were labeled with DAPI (blue staining; left panels). Results are representative of 3 experiments. Original magnification 63x. **C.** DSTN protein expression in normal human skin was assessed by IF using a polyclonal rabbit anti-DSTN antibody (green staining; upper panels) or normal rabbit IgG as a negative control (lower panels). Nuclei were labeled with DAPI (blue staining; left panels). Results are representative of 3 experiments. Dotted lines outline the epidermal-dermal border. Original magnification 63x.
(PPTX)

S7 Fig Localization of GAPDH, DSTN and F-actin in NHK/38 cells. **A.** Localization of GAPDH (red staining; upper left and right panels) and DSTN (green staining; upper middle and right panels) was examined by confocal IF microscopy in 24h Dox-treated NHK/38 cells. Overlap between the red and green fluorescence signals is visible in yellow in the merged image (upper right panel). Co-localization between GAPDH and DSTN is illustrated in white in the co-localization channel (lower left panel). **B.** Localization of DSTN (red staining; upper left and right panels) and F-actin (green staining; upper middle and right panels) was examined by confocal IF microscopy in 24h Dox-treated NHK/38 cells. Overlap between the red and green fluorescence signals is visible in yellow in the merged image (upper right panel). Co-localization between DSTN and F-actin is illustrated in white (co-localization channel; lower left panel). Results are representative of 2 (GAPDH/DSTN) or 1 (DSTN/F-actin) experiment(s). Original magnification 63x.
(PPTX)

S1 Images Original uncropped and unadjusted images of Western blots. This file includes the original uncropped and unadjusted images of the Western blots shown in Fig 2A, Fig 2B and S1B Fig.
(PDF)

## References

[1] Garlanda C, Dinarello CA, Mantovani A. The interleukin-1 family: back to the future. Immunity. 2013;39(6):1003–18. Epub 2013/12/18. 10.1016/j.immuni.2013.11.010 24332029 PMC3933951

[2] Palomo J, Dietrich D, Martin P, Palmer G, Gabay C. The interleukin (IL)-1 cytokine family—Balance between agonists and antagonists in inflammatory diseases. Cytokine. 2015;76(1):25–37. Epub 2015/07/18. 10.1016/j.cyto.2015.06.017 . 26185894

[3] Boraschi D, Italiani P, Weil S, Martin MU. The family of the interleukin-1 receptors. Immunological reviews. 2018;281(1):197–232. Epub 2017/12/17. 10.1111/imr.12606 . 29248002

[4] Lin H, Ho AS, Haley-Vicente D, Zhang J, Bernal-Fussell J, Pace AM, et al Cloning and characterization of IL-1HY2, a novel interleukin-1 family member. The Journal of biological chemistry. 2001;276(23):20597–602. Epub 2001/03/30. 10.1074/jbc.M010095200 . 11278614

[5] van de Veerdonk FL, Stoeckman AK, Wu G, Boeckermann AN, Azam T, Netea MG, et al IL-38 binds to the IL-36 receptor and has biological effects on immune cells similar to IL-36 receptor antagonist. Proceedings of the National Academy of Sciences of the United States of America. 2012;109(8):3001–5. Epub 2012/02/09. 10.1073/pnas.1121534109 22315422 PMC3286950

[6] Rudloff I, Godsell J, Nold-Petry CA, Harris J, Hoi A, Morand EF, et al Brief Report: Interleukin-38 Exerts Antiinflammatory Functions and Is Associated With Disease Activity in Systemic Lupus Erythematosus. Arthritis Rheumatol. 2015;67(12):3219–25. Epub 2015/09/01. 10.1002/art.39328 . 26314375

[7] Boutet MA, Najm A, Bart G, Brion R, Touchais S, Trichet V, et al IL-38 overexpression induces anti-inflammatory effects in mice arthritis models and in human macrophages in vitro. Annals of the rheumatic diseases. 2017 Epub 2017/03/16. 10.1136/annrheumdis-2016-210630 . 28288964

[8] Mora J, Schlemmer A, Wittig I, Richter F, Putyrski M, Frank AC, et al Interleukin-38 is released from apoptotic cells to limit inflammatory macrophage responses. J Mol Cell Biol. 2016 Epub 2016/02/20. 10.1093/jmcb/mjw006 . 26892022

[9] Mercurio L, Morelli M, Scarponi C, Eisenmesser EZ, Doti N, Pagnanelli G, et al IL-38 has an anti-inflammatory action in psoriasis and its expression correlates with disease severity and therapeutic response to anti-IL-17A treatment. Cell death & disease. 2018;9(11):1104 Epub 2018/11/01. 10.1038/s41419-018-1143-3 30377293 PMC6207563

[10] Yuan X, Li Y, Pan X, Peng X, Song G, Jiang W, et al IL-38 alleviates concanavalin A-induced liver injury in mice. International immunopharmacology. 2016;40:452–7. Epub 2016/10/11. 10.1016/j.intimp.2016.09.023 . 27723569

[11] Chu M, Tam LS, Zhu J, Jiao D, Liu H, Cai Z, et al In vivo anti-inflammatory activities of novel cytokine IL-38 in Murphy Roths Large (MRL)/lpr mice. Immunobiology. 2017;222(3):483–93. Epub 2016/10/23. 10.1016/j.imbio.2016.10.012 . 27769564

[12] Xu F, Lin S, Yan X, Wang C, Tu H, Yin Y, et al Interleukin 38 Protects Against Lethal Sepsis. The Journal of infectious diseases. 2018;218(7):1175–84. Epub 2018/05/16. 10.1093/infdis/jiy289 . 29762676

[13] Xu K, Sun J, Chen S, Li Y, Peng X, Li M, et al Hydrodynamic delivery of IL-38 gene alleviates obesity-induced inflammation and insulin resistance. Biochemical and biophysical research communications. 2019;508(1):198–202. Epub 2018/11/28. 10.1016/j.bbrc.2018.11.114 . 30477747

[14] Takenaka S, Kaieda S, Kawayama T, Matsuoka M, Kaku Y, Kinoshita T, et al IL-38: A new factor in rheumatoid arthritis. Biochemistry and Biophysics Reports. 2015;4:386–91. 10.1016/j.bbrep.2015.10.015 29124228 PMC5669445

[15] Ciccia F, Accardo-Palumbo A, Alessandro R, Alessandri C, Priori R, Guggino G, et al Interleukin-36alpha axis is modulated in patients with primary Sjogren's syndrome. Clin Exp Immunol. 2015;181(2):230–8. Epub 2015/04/24. 10.1111/cei.12644 25902739 PMC4516438

[16] Boutet MA, Bart G, Penhoat M, Amiaud J, Brulin B, Charrier C, et al Distinct expression of interleukin (IL)-36alpha, beta and gamma, their antagonist IL-36Ra and IL-38 in psoriasis, rheumatoid arthritis and Crohn's disease. Clin Exp Immunol. 2016;184(2):159–73. Epub 2015/12/25. 10.1111/cei.12761 26701127 PMC4837235

[17] Wang M, Wang B, Ma Z, Sun X, Tang Y, Li X, et al Detection of the novel IL-1 family cytokines by QAH-IL1F-1 assay in rheumatoid arthritis. Cell Mol Biol (Noisy-le-grand). 2016;62(4):31–4. Epub 2016/05/18. . 27188731

[18] Keermann M, Koks S, Reimann E, Abram K, Erm T, Silm H, et al Expression of IL-36 family cytokines and IL-37 but not IL-38 is altered in psoriatic skin. J Dermatol Sci. 2015;80(2):150–2. Epub 2015/09/01. 10.1016/j.jdermsci.2015.08.002 . 26319074

[19] Hessam S, Sand M, Gambichler T, Skrygan M, Ruddel I, Bechara FG. Interleukin-36 in hidradenitis suppurativa: evidence for a distinctive proinflammatory role and a key factor in the development of an inflammatory loop. The British journal of dermatology. 2018;178(3):761–7. Epub 2017/10/05. 10.1111/bjd.16019 . 28975626

[20] Xu WD, Su LC, He CS, Huang AF. Plasma interleukin-38 in patients with rheumatoid arthritis. International immunopharmacology. 2018;65:1–7. Epub 2018/09/30. 10.1016/j.intimp.2018.09.028 . 30268016

[21] Fonseca-Camarillo G, Furuzawa-Carballeda J, Iturriaga-Goyon E, Yamamoto-Furusho JK. Differential Expression of IL-36 Family Members and IL-38 by Immune and Nonimmune Cells in Patients with Active Inflammatory Bowel Disease. BioMed research international. 2018;2018:5140691 Epub 2019/01/16. 10.1155/2018/5140691 30643810 PMC6311241

[22] Chou CT, Timms AE, Wei JC, Tsai WC, Wordsworth BP, Brown MA. Replication of association of IL1 gene complex members with ankylosing spondylitis in Taiwanese Chinese. Annals of the rheumatic diseases. 2006;65(8):1106–9. Epub 2005/12/20. 10.1136/ard.2005.046847 16361275 PMC1798239

[23] Rahman P, Sun S, Peddle L, Snelgrove T, Melay W, Greenwood C, et al Association between the interleukin-1 family gene cluster and psoriatic arthritis. Arthritis and rheumatism. 2006;54(7):2321–5. Epub 2006/08/19. 10.1002/art.21928 . 16918024

[24] Guo ZS, Li C, Lin ZM, Huang JX, Wei QJ, Wang XW, et al Association of IL-1 gene complex members with ankylosing spondylitis in Chinese Han population. International journal of immunogenetics. 2010;37(1):33–7. Epub 2009/11/26. 10.1111/j.1744-313X.2009.00889.x . 19930406

[25] Jung MY, Kang SW, Kim SK, Kim HJ, Yun DH, Yim SV, et al The interleukin-1 family gene polymorphisms in Korean patients with rheumatoid arthritis. Scandinavian journal of rheumatology. 2010;39(3):190–6. Epub 2010/02/10. 10.3109/03009740903447028 . 20141484

[26] Dehghan A, Dupuis J, Barbalic M, Bis JC, Eiriksdottir G, Lu C, et al Meta-analysis of genome-wide association studies in >80 000 subjects identifies multiple loci for C-reactive protein levels. Circulation. 2011;123(7):731–8. Epub 2011/02/09. 10.1161/CIRCULATIONAHA.110.948570 21300955 PMC3147232

[27] Monnet D, Kadi A, Izac B, Lebrun N, Letourneur F, Zinovieva E, et al Association between the IL-1 family gene cluster and spondyloarthritis. Annals of the rheumatic diseases. 2012;71(6):885–90. Epub 2012/02/09. 10.1136/annrheumdis-2011-200439 . 22312160

[28] Soto Lopez ME, Gamboa Avila R, Hernandez E, Huesca-Gomez C, Castrejon-Tellez V, Perez-Mendez O, et al The interleukin-1 gene cluster polymorphisms are associated with Takayasu's arteritis in Mexican patients. Journal of interferon & cytokine research: the official journal of the International Society for Interferon and Cytokine Research. 2013;33(7):369–75. Epub 2013/03/12. 10.1089/jir.2012.0126 . 23472661

[29] Ligthart S, de Vries PS, Uitterlinden AG, Hofman A, Franco OH, Chasman DI, et al Pleiotropy among common genetic loci identified for cardiometabolic disorders and C-reactive protein. PloS one. 2015;10(3):e0118859 Epub 2015/03/15. 10.1371/journal.pone.0118859 ; PubMed Central PMCID: PMC4358943. 25768928 PMC4358943

[30] Lachner J, Mlitz V, Tschachler E, Eckhart L. Epidermal cornification is preceded by the expression of a keratinocyte-specific set of pyroptosis-related genes. Scientific reports. 2017;7(1):17446 Epub 2017/12/14. 10.1038/s41598-017-17782-4 29234126 PMC5727156

[31] Rider P, Carmi Y, Voronov E, Apte RN. Interleukin-1alpha. Seminars in immunology. 2013;25(6):430–8. Epub 2013/11/05. 10.1016/j.smim.2013.10.005 . 24183701

[32] Steenbergen RD, Walboomers JM, Meijer CJ, van der Raaij-Helmer EM, Parker JN, Chow LT, et al Transition of human papillomavirus type 16 and 18 transfected human foreskin keratinocytes towards immortality: activation of telomerase and allele losses at 3p, 10p, 11q and/or 18q. Oncogene. 1996;13(6):1249–57. Epub 1996/09/19. . 8808699

[33] Palomo J, Troccaz S, Talabot-Ayer D, Rodriguez E, Palmer G. The severity of imiquimod-induced mouse skin inflammation is independent of endogenous IL-38 expression. PloS one. 2018;13(3):e0194667 Epub 2018/03/20. 10.1371/journal.pone.0194667 . 29554104 PMC5858842

[34] Carta S, Lavieri R, Rubartelli A. Different Members of the IL-1 Family Come Out in Different Ways: DAMPs vs. Cytokines? Frontiers in immunology. 2013;4:123 Epub 2013/06/08. 10.3389/fimmu.2013.00123 23745123 PMC3662868

[35] Van Opdenbosch N, Lamkanfi M. Caspases in Cell Death, Inflammation, and Disease. Immunity. 2019;50(6):1352–64. Epub 2019/06/20. 10.1016/j.immuni.2019.05.020 31216460 PMC6611727

[36] Rider P, Voronov E, Dinarello CA, Apte RN, Cohen I. Alarmins: Feel the Stress. Journal of immunology (Baltimore, Md: 1950). 2017;198(4):1395–402. Epub 2017/02/09. 10.4049/jimmunol.1601342 . 28167650

[37] Haskill S, Martin G, Van Le L, Morris J, Peace A, Bigler CF, et al cDNA cloning of an intracellular form of the human interleukin 1 receptor antagonist associated with epithelium. Proceedings of the National Academy of Sciences of the United States of America. 1991;88(9):3681–5. Epub 1991/05/01. 10.1073/pnas.88.9.3681 1827201 PMC51516

[38] Lee RT, Briggs WH, Cheng GC, Rossiter HB, Libby P, Kupper T. Mechanical deformation promotes secretion of IL-1 alpha and IL-1 receptor antagonist. Journal of immunology (Baltimore, Md: 1950). 1997;159(10):5084–8. Epub 1997/11/20. . 9366437

[39] Mee JB, Antonopoulos C, Poole S, Kupper TS, Groves RW. Counter-regulation of interleukin-1alpha (IL-1alpha) and IL-1 receptor antagonist in murine keratinocytes. The Journal of investigative dermatology. 2005;124(6):1267–74. Epub 2005/06/16. 10.1111/j.0022-202X.2005.23684.x . 15955103

[40] Afonina IS, Muller C, Martin SJ, Beyaert R. Proteolytic Processing of Interleukin-1 Family Cytokines: Variations on a Common Theme. Immunity. 2015;42(6):991–1004. Epub 2015/06/18. 10.1016/j.immuni.2015.06.003 . 26084020

[41] Garraud T, Harel M, Boutet MA, Le Goff B, Blanchard F. The enigmatic role of IL-38 in inflammatory diseases. Cytokine & growth factor reviews. 2018;39:26–35. Epub 2018/01/26. 10.1016/j.cytogfr.2018.01.001 . 29366546

[42] Fields JK, Gunther S, Sundberg EJ. Structural Basis of IL-1 Family Cytokine Signaling. Frontiers in immunology. 2019;10:1412 Epub 2019/07/10. 10.3389/fimmu.2019.01412 31281320 PMC6596353

[43] Han Y, Mora J, Huard A, da Silva P, Wiechmann S, Putyrski M, et al IL-38 Ameliorates Skin Inflammation and Limits IL-17 Production from gammadelta T Cells. Cell reports. 2019;27(3):835–46.e5. Epub 2019/04/18. 10.1016/j.celrep.2019.03.082 . 30995480

[44] Kanellos G, Zhou J, Patel H, Ridgway RA, Huels D, Gurniak CB, et al ADF and Cofilin1 Control Actin Stress Fibers, Nuclear Integrity, and Cell Survival. Cell reports. 2015;13(9):1949–64. Epub 2015/12/15. 10.1016/j.celrep.2015.10.056 26655907 PMC4678118

[45] Yin H, Morioka H, Towle CA, Vidal M, Watanabe T, Weissbach L. Evidence that HAX-1 is an interleukin-1 alpha N-terminal binding protein. Cytokine. 2001;15(3):122–37. Epub 2001/09/14. 10.1006/cyto.2001.0891 . 11554782

[46] Hu B, Wang S, Zhang Y, Feghali CA, Dingman JR, Wright TM. A nuclear target for interleukin-1alpha: interaction with the growth suppressor necdin modulates proliferation and collagen expression. Proceedings of the National Academy of Sciences of the United States of America. 2003;100(17):10008–13. Epub 2003/08/13. 10.1073/pnas.1737765100 12913118 PMC187743

[47] Banda NK, Guthridge C, Sheppard D, Cairns KS, Muggli M, Bech-Otschir D, et al Intracellular IL-1 receptor antagonist type 1 inhibits IL-1-induced cytokine production in keratinocytes through binding to the third component of the COP9 signalosome. Journal of immunology (Baltimore, Md: 1950). 2005;174(6):3608–16. Epub 2005/03/08. 10.4049/jimmunol.174.6.3608 . 15749898

[48] Nold MF, Nold-Petry CA, Zepp JA, Palmer BE, Bufler P, Dinarello CA. IL-37 is a fundamental inhibitor of innate immunity. Nature immunology. 2010;11(11):1014–22. Epub 2010/10/12. 10.1038/ni.1944 20935647 PMC3537119

[49] Verdoni AM, Smith RS, Ikeda A, Ikeda S. Defects in actin dynamics lead to an autoinflammatory condition through the upregulation of CXCL5. PloS one. 2008;3(7):e2701 Epub 2008/07/17. 10.1371/journal.pone.0002701 18628996 PMC2442876

[50] Vaezi A, Bauer C, Vasioukhin V, Fuchs E. Actin cable dynamics and Rho/Rock orchestrate a polarized cytoskeletal architecture in the early steps of assembling a stratified epithelium. Developmental cell. 2002;3(3):367–81. Epub 2002/10/04. 10.1016/s1534-5807(02)00259-9 . 12361600

