# Supplementary figures and images for: Interleukin-38 interacts with destrin/actin-depolymerizing factor in human keratinocytes
Source: PLoS One. 2019 Nov 26;14(11):e0225782. doi: 10.1371/journal.pone.0225782 (PMC6879167; doi:10.1371/journal.pone.0225782)

## Slide 1
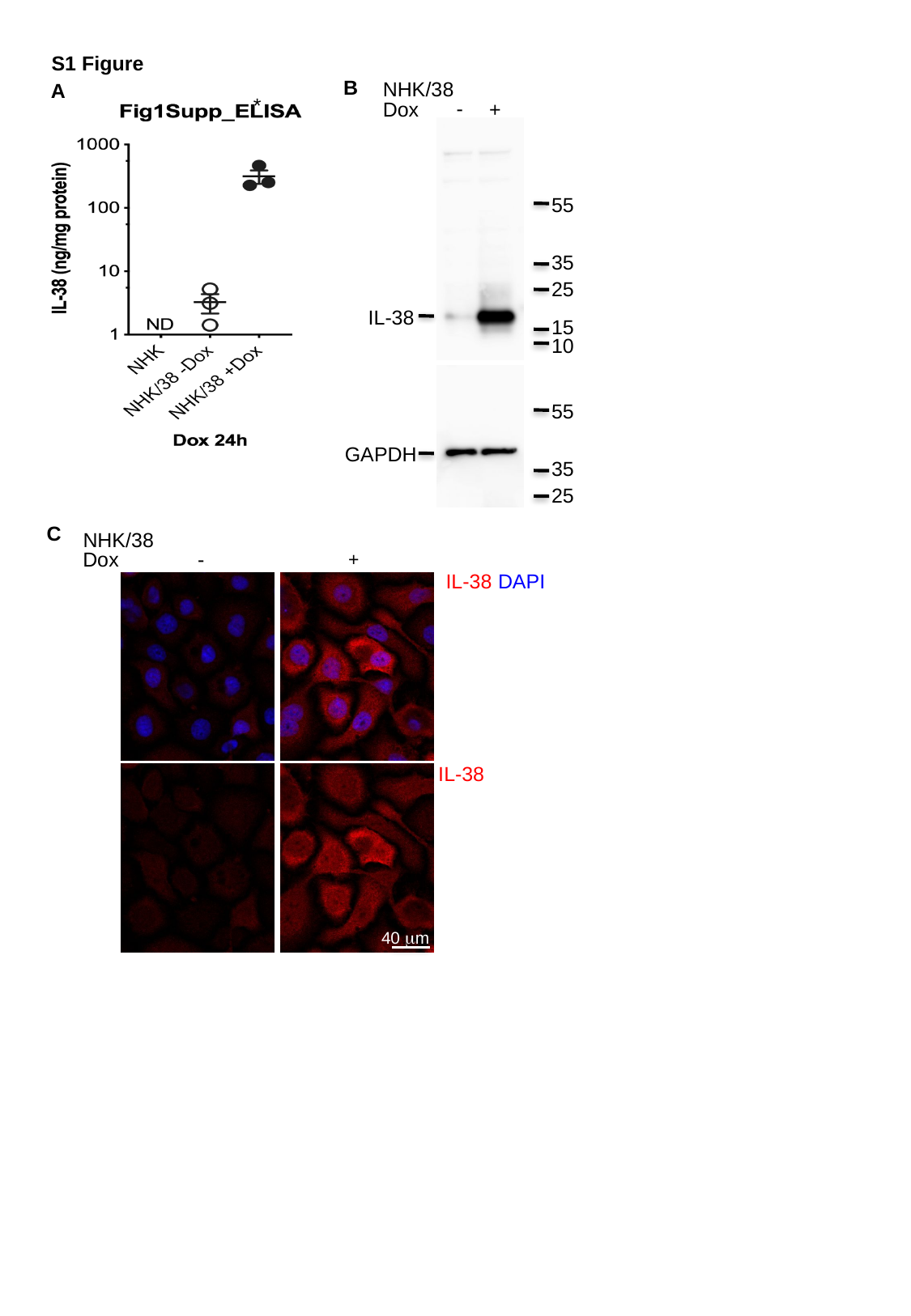

S1 Figure
NHK/38
 B
 A
*
-
Dox
+
55
35
25
IL-38
15
10
55
GAPDH
35
25
C
NHK/38
Dox
-
+
IL-38 DAPI
IL-38
40 mm

Supplement: S1 Fig — A. IL-38 protein levels in NHK/38 cell lysates after 24h incubation without or with Dox were assessed by ELISA. Results are expressed as ng IL-38 per mg of cellular protein. Data are shown as individual values and mean ± SEM for triplicate wells in one experiment representative of 4 independent cultures. *p<0.05 vs. NHK and NHK/38 -Dox cells, as assessed by one-way ANOVA, followed by Tukey’s multiple comparisons test. B. IL-38 protein expression in NHK/38 cells without or with 24h Dox treatment was assessed by Western blotting (upper panel). The membrane was then stripped and reprobed with an anti-GAPDH antibody as a loading control (lower panel). Results are representative of 2 experiments. C. IL-38 protein expression in NHK/38 cells without (left panels) or with (right panels) 24h Dox treatment was assessed by IF (red staining; all panels). Nuclei were labeled with DAPI (blue staining; upper panels). Results are representative of 5 independent experiments. Original magnification 63x. (PPTX) [file pone.0225782.s001.pptx]

## Slide 1
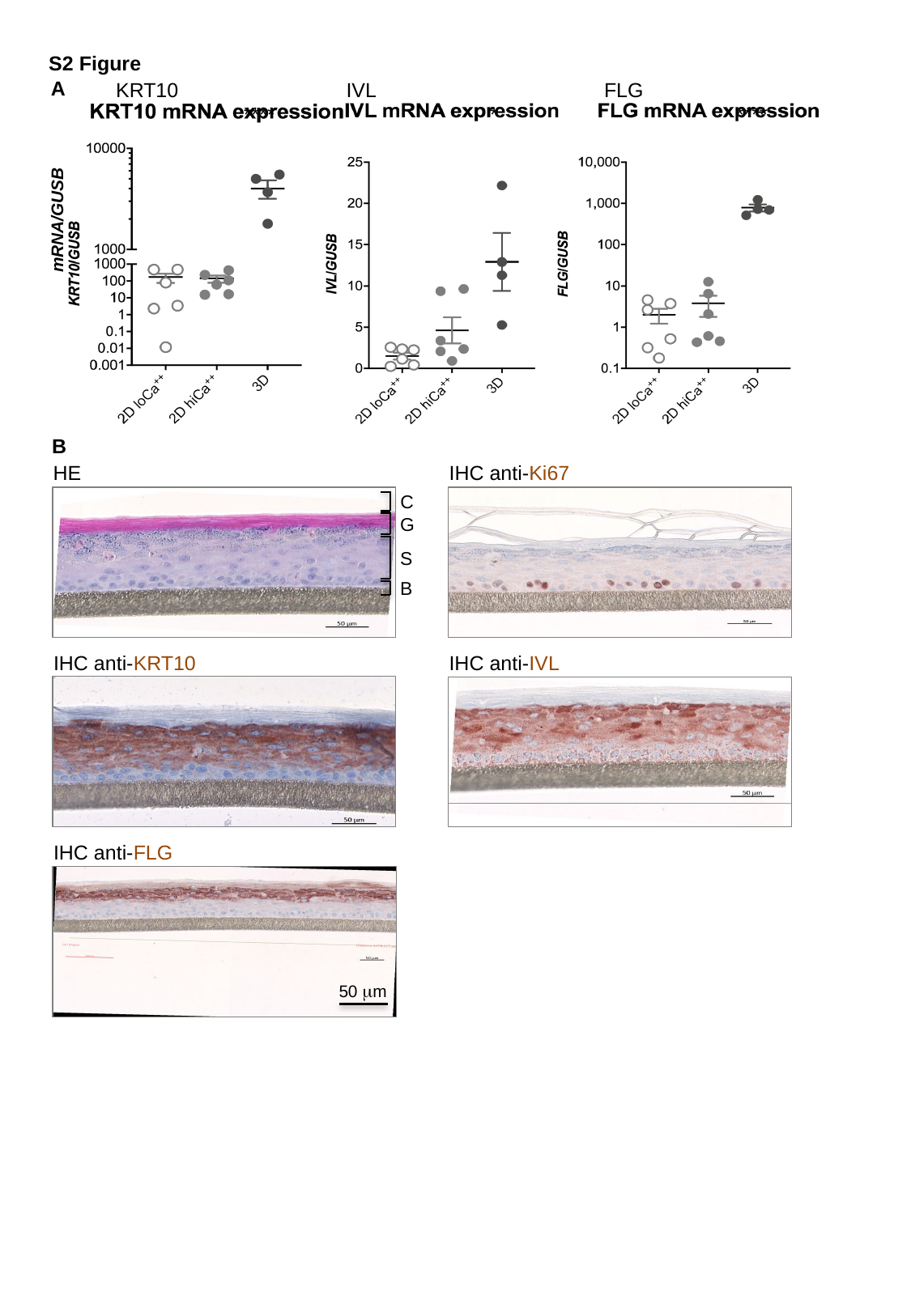

S2 Figure
KRT10
IVL
FLG
 A
****
*
****
mRNA/GUSB
 B
HE
IHC anti-Ki67
C
G
S
B
IHC anti-KRT10
IHC anti-IVL
IHC anti-FLG
50 mm

Supplement: S2 Fig — A. KRT10 (left panel), IVL (middle panel) and FLG (right panel) mRNA levels were assessed by RT-qPCR in primary human keratinocytes cultured in monolayers (2D) in presence of low (lo; 0.06mM) or high (hi; 2mM) Ca++, or in RHE. Transcript levels are expressed relative to GUSB. Results are shown as individual values and mean ± SEM for 6 (2D cultures) or 4 (RHE) different donors. *p<0.05, ****p>0.0001 vs. 2D cultures, as assessed by one-way ANOVA, followed by Tukey’s multiple comparisons test. B. The structure of the RHE was examined by HE staining (upper left panel). After 10 days of culture at the air-liquid interface, the cells formed a morphologically well-defined epidermis-like structure with (from bottom to top) basal (B), spinous (S), granular (G) and cornified (C) layers similar to in vivo skin. Protein expression of keratinocyte proliferation (Ki67; brown staining, upper right panel) and differentiation (KRT10, IVL, FLG; brown staining, lower panels) markers was assessed by IHC. Original magnification 10x. (PPTX) [file pone.0225782.s002.pptx]
