# Supplementary material for: Interleukin-38 interacts with destrin/actin-depolymerizing factor in human keratinocytes
Source: PLoS One. 2019 Nov 26;14(11):e0225782. doi: 10.1371/journal.pone.0225782 (PMC6879167; doi:10.1371/journal.pone.0225782)
Supplement: S3 Fig — IL-38 was detected by IF in HEK 293T cells transfected with pcDNA3.1/hIL-38 (red staining, overexpressed IL-38; upper panels) or with empty pcDNA3.1 as a negative control (lower panels) using the AF2427 polyclonal goat anti-IL-38 antibody (A) or the H127C monoclonal mouse anti-IL-38 antibody (B). IL-38 was detected by IF in 24h Dox-treated NHK/38 cells (red staining, overexpressed IL-38; upper panels) or NHK/lacZ cells used as a negative control (lower panels) using the AF2427 polyclonal goat anti-IL-38 antibody (C) or the H127C monoclonal mouse anti-IL-38 antibody (D). Nuclei were labeled with DAPI (blue staining; left panels). Original magnification 40x. (PPTX) [file pone.0225782.s003.pptx]

## Slide 1
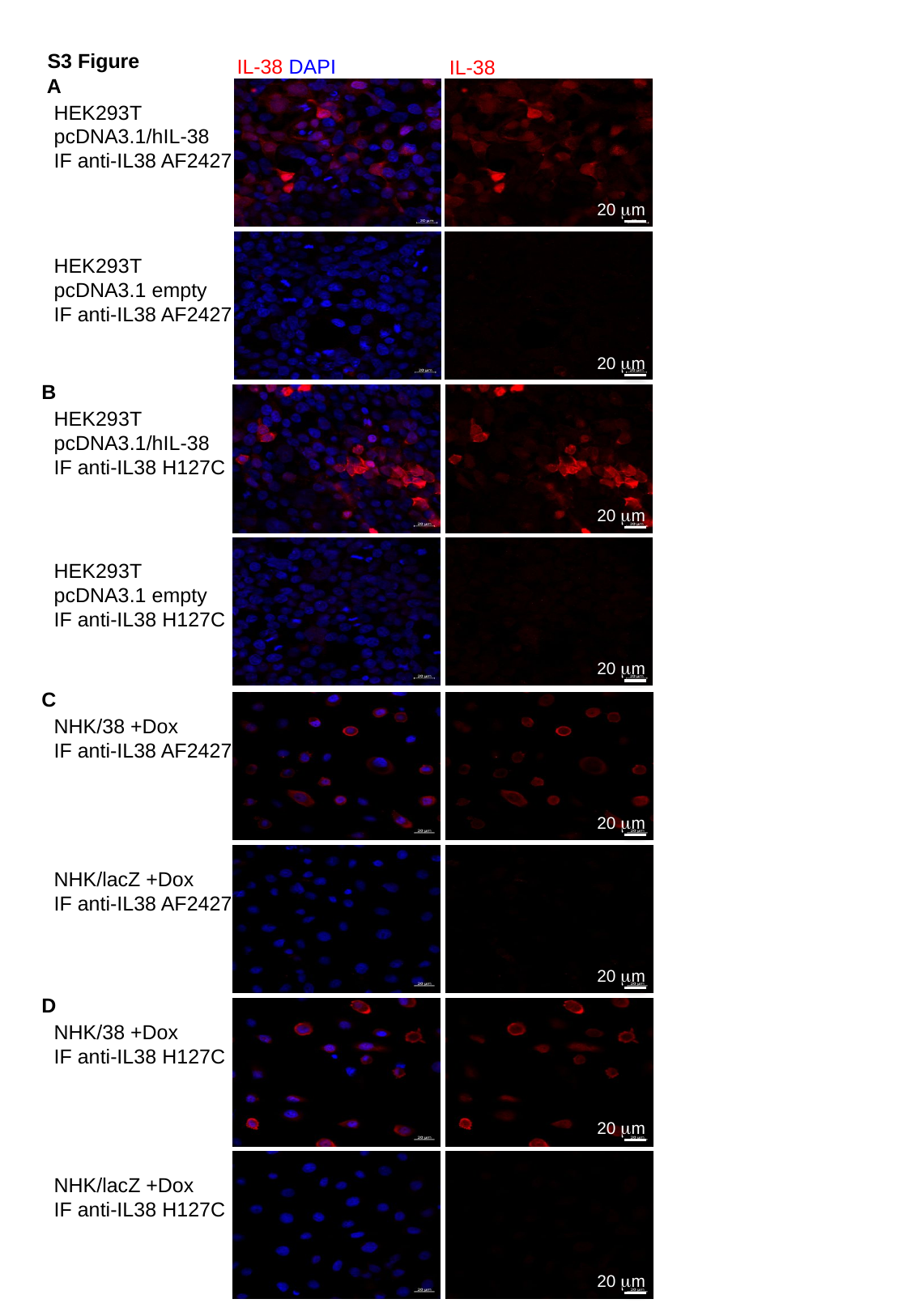

IL-38 DAPI
IL-38
 S3 Figure
 A
HEK293T
pcDNA3.1/hIL-38
IF anti-IL38 AF2427
20 mm
HEK293T
pcDNA3.1 empty
IF anti-IL38 AF2427
20 mm
B
HEK293T
pcDNA3.1/hIL-38
IF anti-IL38 H127C
20 mm
HEK293T
pcDNA3.1 empty
IF anti-IL38 H127C
20 mm
C
NHK/38 +Dox
IF anti-IL38 AF2427
20 mm
NHK/lacZ +Dox
IF anti-IL38 AF2427
20 mm
D
NHK/38 +Dox
IF anti-IL38 H127C
20 mm
NHK/lacZ +Dox
IF anti-IL38 H127C
20 mm
