# Supplementary material for: Interleukin-38 interacts with destrin/actin-depolymerizing factor in human keratinocytes
Source: PLoS One. 2019 Nov 26;14(11):e0225782. doi: 10.1371/journal.pone.0225782 (PMC6879167; doi:10.1371/journal.pone.0225782)
Supplement: S4 Fig — A. IL-38 was detected by IF in RHE using a monoclonal mouse anti-IL-38 antibody (red staining; upper panels) or normal mouse IgG as a negative control (lower panels). Nuclei were labeled with DAPI (blue staining; left panels). Results are representative of 5 independent experiments. Original magnification 63x. B. IL-38 protein expression in RHE was examined by IF using a monoclonal mouse anti-IL-38 antibody (red staining; upper panels) or the same antibody pre-adsorbed with recombinant human IL-38 (lower panels). Nuclei were labeled with DAPI (blue staining; left panels). Results are representative of 2 independent experiments. Original magnification 63x. C. IL-38 protein expression in normal human skin was assessed by IF using a monoclonal mouse anti-IL-38 antibody (red staining; upper panels) or normal mouse IgG as a negative control (lower panels). Nuclei were labeled with DAPI (blue staining; left panels). Results are representative of 3 different donors. Dotted lines outline the epidermal-dermal border. Original magnification 40x. (PPTX) [file pone.0225782.s004.pptx]

## Slide 1
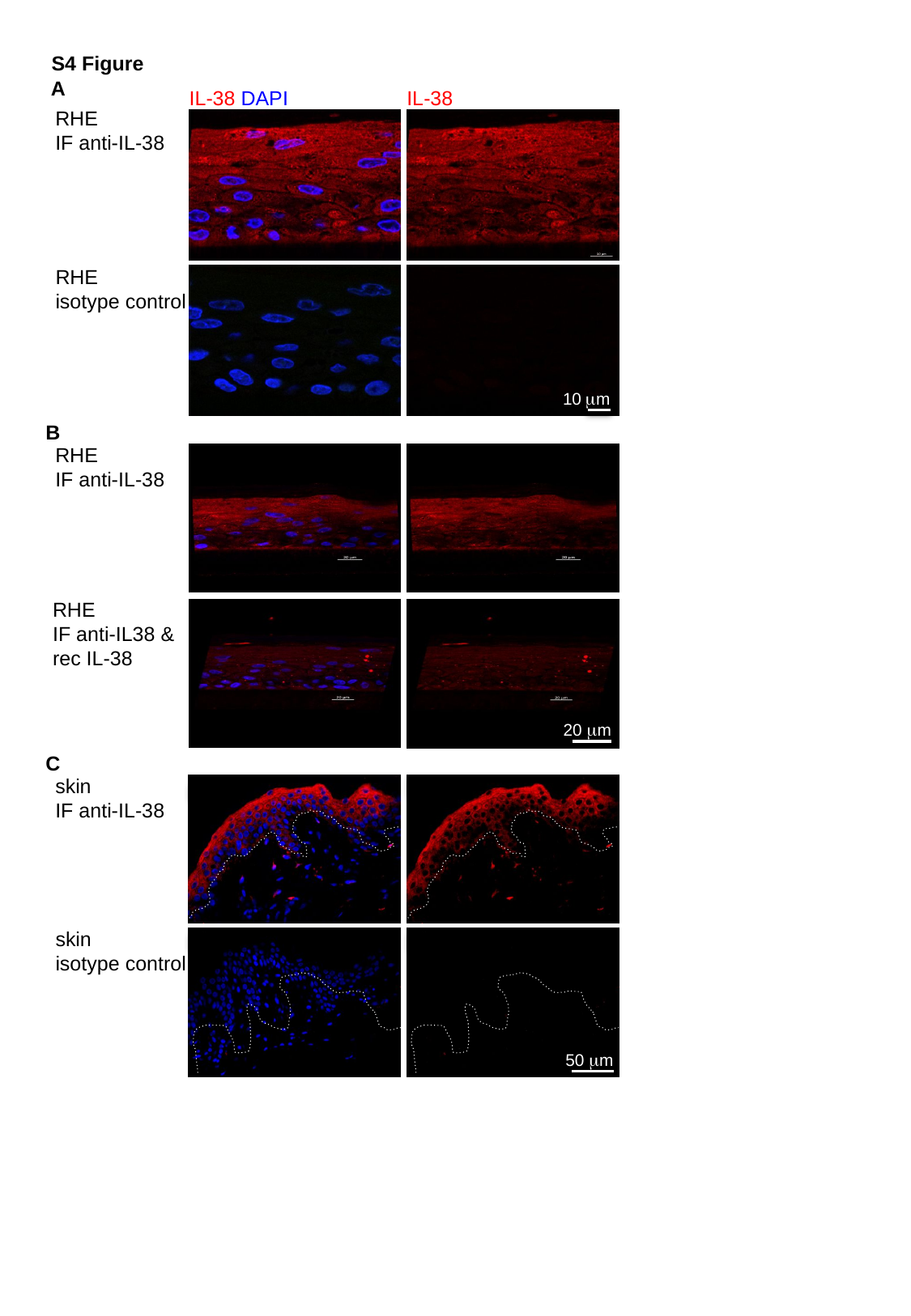

S4 Figure
 A
IL-38 DAPI
IL-38
RHE
IF anti-IL-38
RHE
isotype control
10 mm
B
RHE
IF anti-IL-38
RHE
IF anti-IL38 &
rec IL-38
20 mm
C
skin
IF anti-IL-38
skin
isotype control
50 mm
