# Supplementary material for: Interleukin-38 interacts with destrin/actin-depolymerizing factor in human keratinocytes
Source: PLoS One. 2019 Nov 26;14(11):e0225782. doi: 10.1371/journal.pone.0225782 (PMC6879167; doi:10.1371/journal.pone.0225782)
Supplement: S5 Fig — Negative controls for the PLA experiment were performed by incubation of 24h Dox-treated NHK/38 cells with the anti-DSTN antibody alone (upper panels), the anti-IL-38 antibody alone (middle panels) or antibody diluent only (lower panels). After addition of PLA probes and signal amplification, only minimal background staining was observed (red staining; all panels). Nuclei were labeled with DAPI (blue staining, right panels). Original magnification 63x. (PPTX) [file pone.0225782.s005.pptx]

## Slide 1
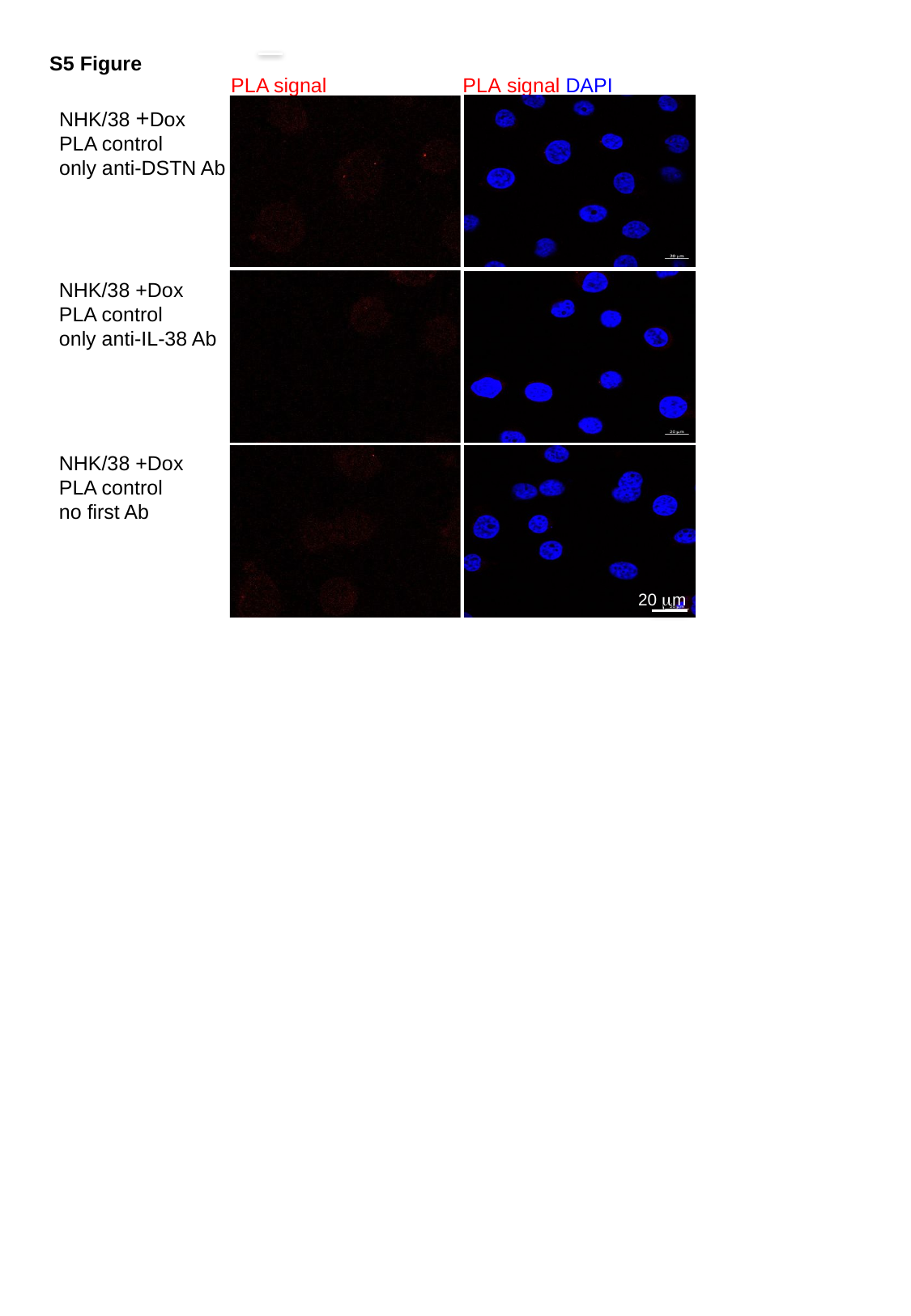

S5 Figure
PLA signal
PLA signal DAPI
NHK/38 +Dox
PLA control
only anti-DSTN Ab
NHK/38 +Dox
PLA control
only anti-IL-38 Ab
NHK/38 +Dox
PLA control
no first Ab
20 mm
