# Supplementary material for: Interleukin-38 interacts with destrin/actin-depolymerizing factor in human keratinocytes
Source: PLoS One. 2019 Nov 26;14(11):e0225782. doi: 10.1371/journal.pone.0225782 (PMC6879167; doi:10.1371/journal.pone.0225782)
Supplement: S6 Fig — A. DSTN was detected by IF in HEK 293T cells transfected with pcDNA3.1/hDSTN (green staining, overexpressed DSTN; upper panels) or empty pcDNA3.1 (green staining, endogenous DSTN; middle panels) using a polyclonal rabbit anti-DSTN antibody. Staining with normal rabbit IgG, used as a negative control, is shown for HEK293T cells transfected with pcDNA3.1/hDSTN (lower panels). Nuclei were labeled with DAPI (blue staining; left panels). Original magnification 20x. B. DSTN was detected by IF in RHE using a polyclonal rabbit anti-DSTN antibody (green staining; upper panels). Detection with the labeled secondary anti-rabbit antibody alone is shown as a negative control (lower panels). Nuclei were labeled with DAPI (blue staining; left panels). Results are representative of 3 experiments. Original magnification 63x. C. DSTN protein expression in normal human skin was assessed by IF using a polyclonal rabbit anti-DSTN antibody (green staining; upper panels) or normal rabbit IgG as a negative control (lower panels). Nuclei were labeled with DAPI (blue staining; left panels). Results are representative of 3 experiments. Dotted lines outline the epidermal-dermal border. Original magnification 63x. (PPTX) [file pone.0225782.s006.pptx]

## Slide 1
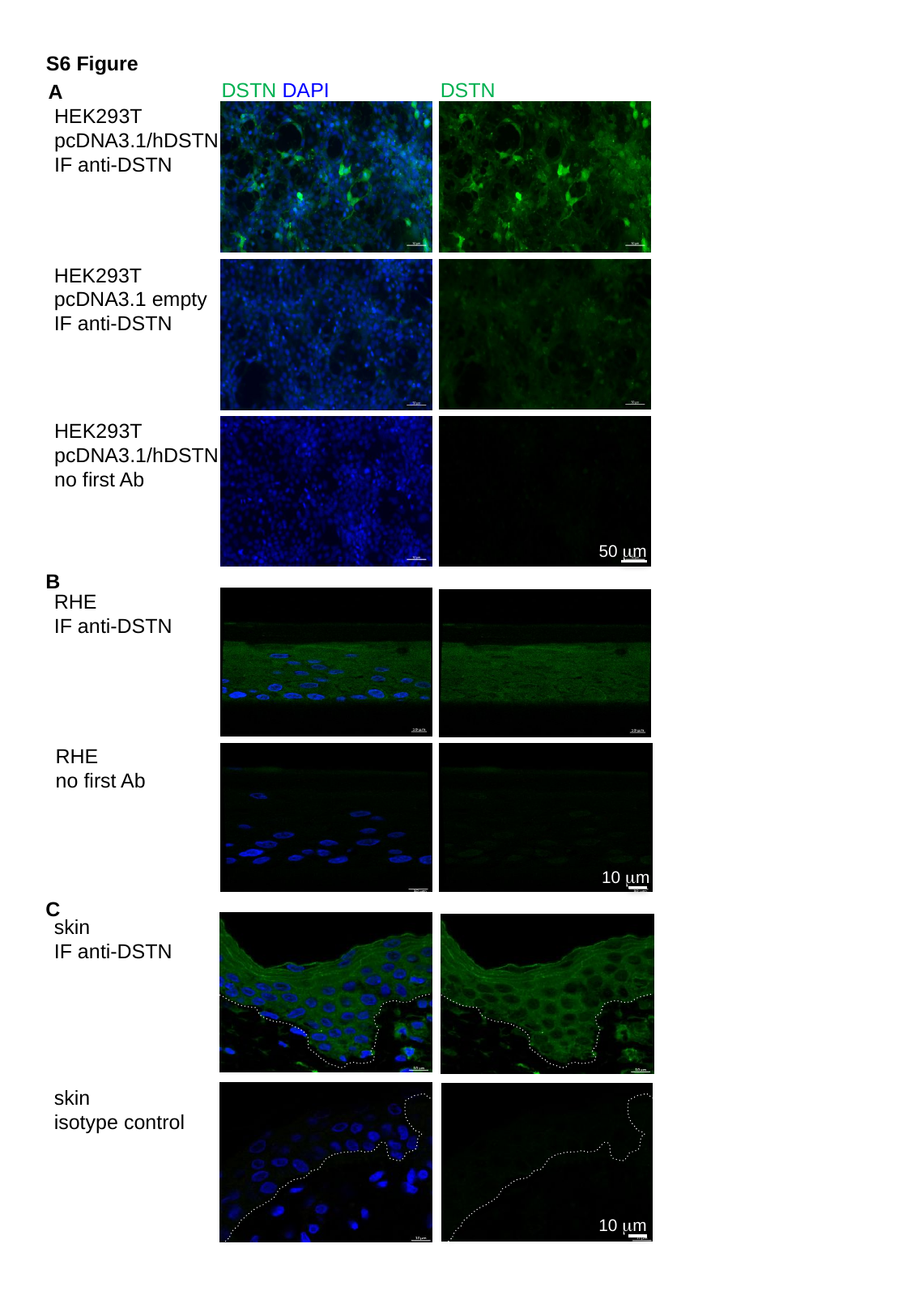

S6 Figure
DSTN DAPI
DSTN
 A
HEK293T
pcDNA3.1/hDSTN
IF anti-DSTN
HEK293T
pcDNA3.1 empty
IF anti-DSTN
HEK293T
pcDNA3.1/hDSTN
no first Ab
50 mm
B
RHE
IF anti-DSTN
RHE
no first Ab
10 mm
C
skin
IF anti-DSTN
skin
isotype control
10 mm
