# Supplementary material for: Interleukin-38 interacts with destrin/actin-depolymerizing factor in human keratinocytes
Source: PLoS One. 2019 Nov 26;14(11):e0225782. doi: 10.1371/journal.pone.0225782 (PMC6879167; doi:10.1371/journal.pone.0225782)
Supplement: S7 Fig — A. Localization of GAPDH (red staining; upper left and right panels) and DSTN (green staining; upper middle and right panels) was examined by confocal IF microscopy in 24h Dox-treated NHK/38 cells. Overlap between the red and green fluorescence signals is visible in yellow in the merged image (upper right panel). Co-localization between GAPDH and DSTN is illustrated in white in the co-localization channel (lower left panel). B. Localization of DSTN (red staining; upper left and right panels) and F-actin (green staining; upper middle and right panels) was examined by confocal IF microscopy in 24h Dox-treated NHK/38 cells. Overlap between the red and green fluorescence signals is visible in yellow in the merged image (upper right panel). Co-localization between DSTN and F-actin is illustrated in white (co-localization channel; lower left panel). Results are representative of 2 (GAPDH/DSTN) or 1 (DSTN/F-actin) experiment(s). Original magnification 63x. (PPTX) [file pone.0225782.s007.pptx]

## Slide 1
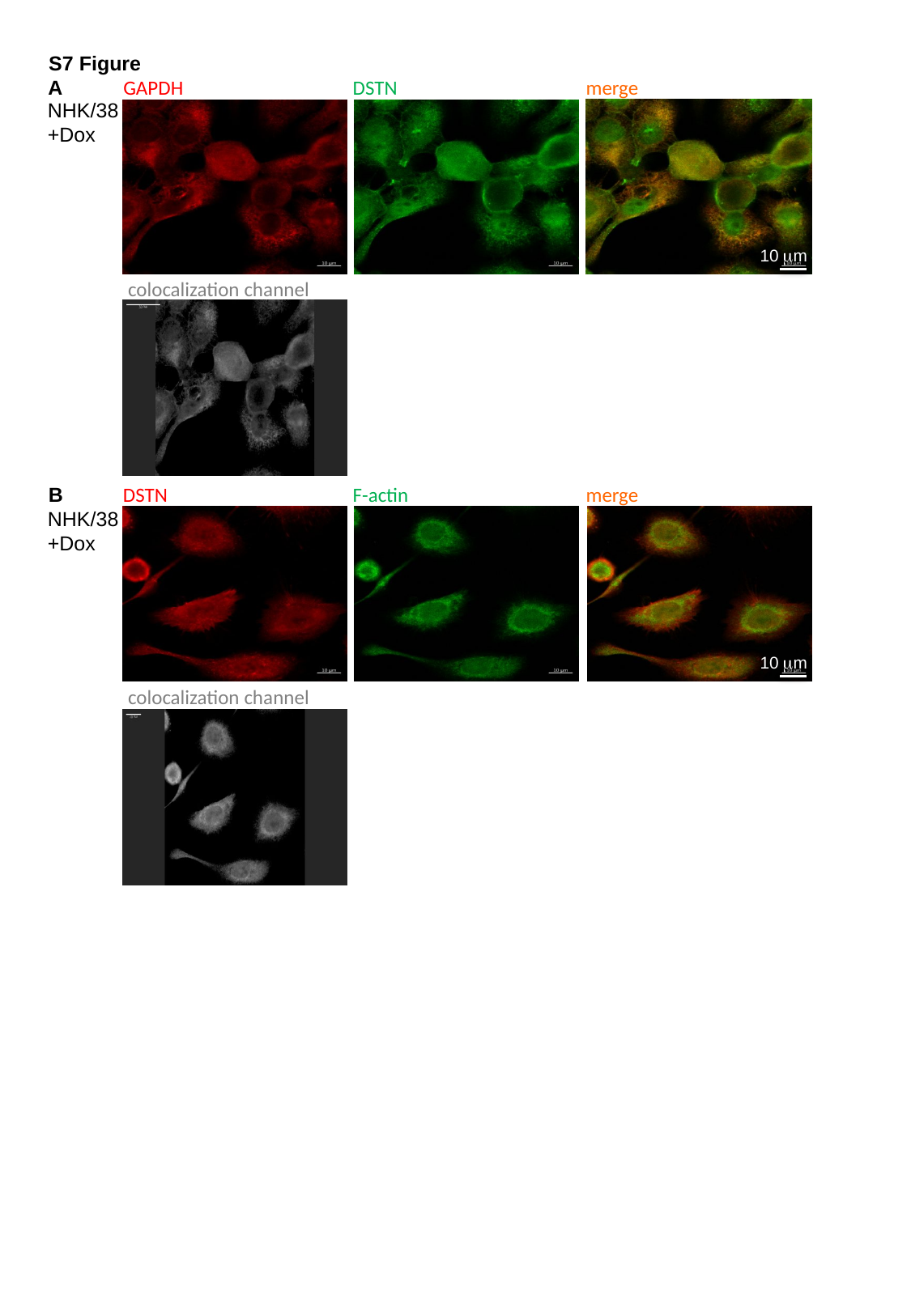

S7 Figure
GAPDH
DSTN
merge
 A
NHK/38
+Dox
10 mm
colocalization channel
DSTN
F-actin
merge
 B
NHK/38
+Dox
10 mm
colocalization channel
