# Supplementary material for: Interleukin-38 interacts with destrin/actin-depolymerizing factor in human keratinocytes
Source: PLoS One. 2019 Nov 26;14(11):e0225782. doi: 10.1371/journal.pone.0225782 (PMC6879167; doi:10.1371/journal.pone.0225782)
Supplement: S1 Images — This file includes the original uncropped and unadjusted images of the Western blots shown in Fig 2A, Fig 2B and S1B Fig. (PDF) [file pone.0225782.s008.pdf]

S1 Images

Original uncropped and unadjusted images of Western blots for Fig 2A

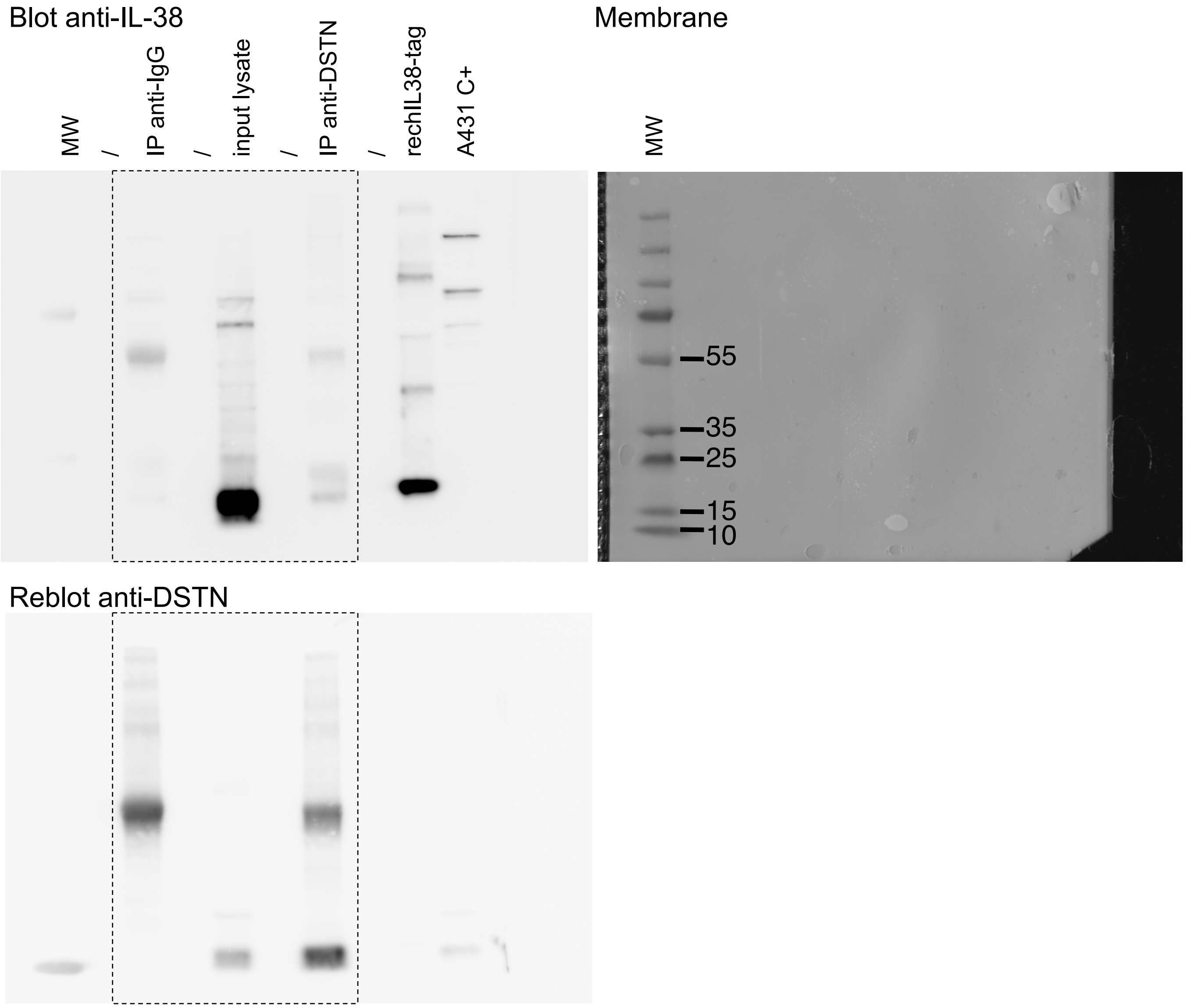

Immunoreactive bands were detected using Radiance Plus chemiluminescent detection on a LAS4000 imager.
Boxes highlight lanes used in Figure 2A.

Original uncropped and unadjusted images of Western blots for Fig 2B

Blot anti-DSTN

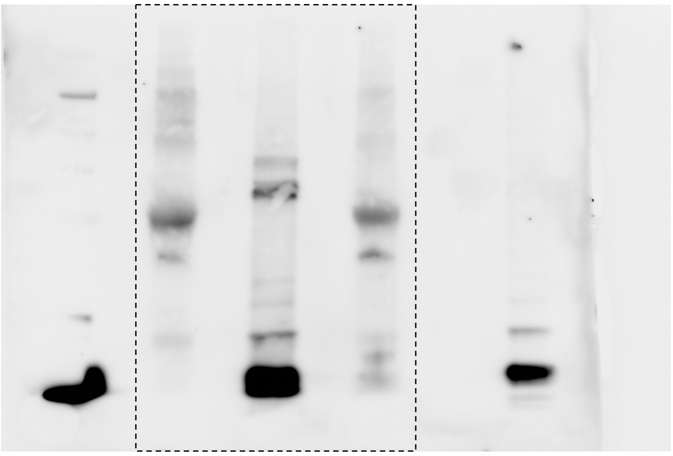

Membrane

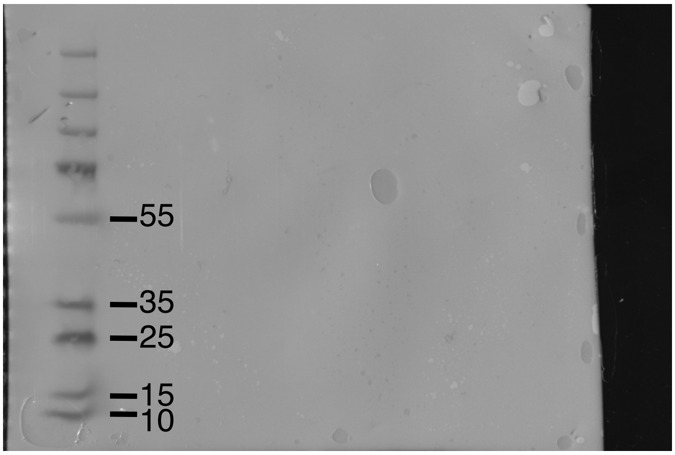

Reblot anti-IL38

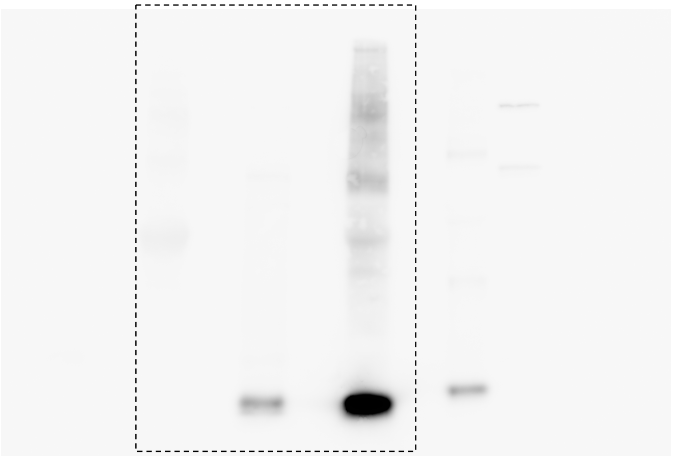

Immunoreactive bands were detected using Radiance Plus chemiluminescent detection on a LAS4000 imager.
Boxes highlight lanes used in Figure 2B.

Original uncropped and unadjusted images of Western blots for S1B Figure

Blot anti-IL-38

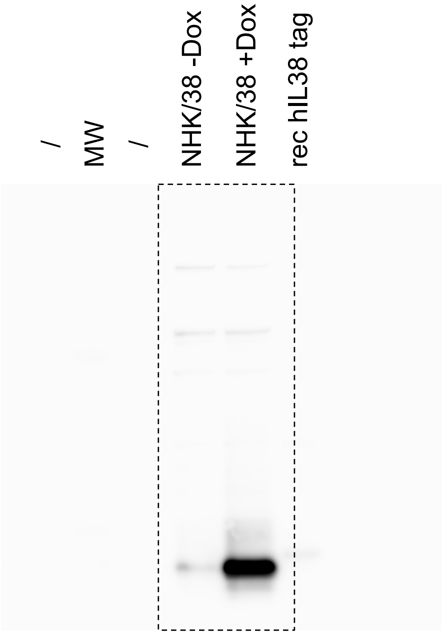

Membrane

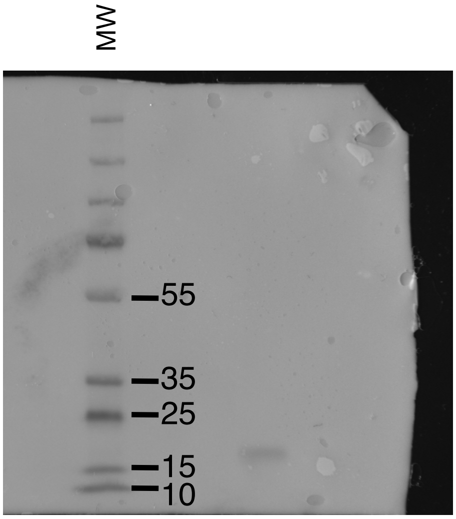

Reblot anti-GAPDH

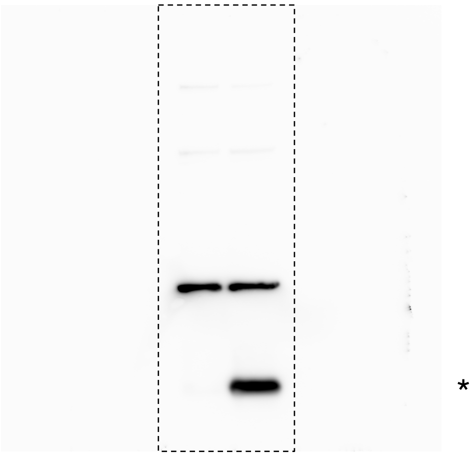

Immunoreactive bands were detected using Radiance Plus chemiluminescent detection on a LAS4000 imager.

Boxes highlight lanes used in S1 Figure, panel B.

*non-specific band due to incomplete stripping of biotinylated anti-IL-38/streptavidin HRP complex
